# Combined metabolomics and network pharmacology to elucidate the mechanisms of Dracorhodin Perchlorate in treating diabetic foot ulcer rats

**DOI:** 10.3389/fphar.2022.1038656

**Published:** 2022-11-18

**Authors:** Pin Deng, Huan Liang, Shulong Wang, Ruinan Hao, Jinglu Han, Xiaojie Sun, Xuyue Pan, Dongxiao Li, Yinwen Wu, Zhichao Huang, Jiajia Xue, Zhaojun Chen

**Affiliations:** ^1^ School of Graduates, Beijing University of Chinese Medicine, Beijing, China; ^2^ Department of Hand and Foot Surgery, Beijing University of Chinese Medicine Third Affiliated Hospital, Beijing, China; ^3^ Department of Orthopedics, Beijing Longfu Hospital, Beijing, China; ^4^ State Key Laboratory of Organic-Inorganic Composites, Beijing University of Chemical Technology, Beijing, China; ^5^ Beijing Laboratory of Biomedical Materials, Beijing University of Chemical Technology, Beijing, China

**Keywords:** diabetic foot ulcer, Dracorhodin Perchlorate, metabolomics, network pharmacology, mechanisms

## Abstract

**Background:** Diabetic foot ulcer (DFU) is a severe chronic complication of diabetes, that can result in disability or death. Dracorhodin Perchlorate (DP) is effective for treating DFU, but the potential mechanisms need to be investigated. We aimed to explore the mechanisms underlying the acceleration of wound healing in DFU by the topical application of DP through the combination of metabolomics and network pharmacology.

**Methods:** A DFU rat model was established, and the rate of ulcer wound healing was assessed. Different metabolites were found in the skin tissues of each group, and MetaboAnalyst was performed to analyse metabolic pathways. The candidate targets of DP in the treatment of DFU were screened using network pharmacology. Cytoscape was applied to construct an integrated network of metabolomics and network pharmacology. Moreover, the obtained hub targets were validated using molecular docking. After the topical application of DP, blood glucose, the rate of wound healing and pro-inflammatory cytokine levels were assessed.

**Results:** The levels of IL-1, hs-CRP and TNF-α of the Adm group were significantly downregulated. A total of 114 metabolites were identified. These could be important to the therapeutic effects of DP in the treatment of DFU. Based on the network pharmacology, seven hub genes were found, which were partially consistent with the metabolomics results. We focused on four hub targets by further integrated analysis, namely, PAH, GSTM1, DHFR and CAT, and the crucial metabolites and pathways. Molecular docking results demonstrated that DP was well combined with the hub targets.

**Conclusion:** Our research based on metabolomics and network pharmacology demonstrated that DP improves wound healing in DFU through multiple targets and pathways, and it can potentially be used for DFU treatment.

## 1 Introduction

Diabetic foot ulcer (DFU), which can cause disability or death in severe cases, is a common chronic complication of diabetes mellitus (DM) (Deng et al., 2022). Reportedly, 19%–34% of people would be afflicted with DFU in a lifetime (Bonnet JB, 2021). DFU is the cause of 50%–70% of limb amputations. One leg is amputated due to DFU every 30 s in the world (Alven et al., 2022). Most wound dressing scaffolds currently in use are ineffective for treating DFU. Amputations are common when ulcer wounds are not adequately treated. The International Working Group on the Diabetic Foot (IWGDF) indicated that the severity of the diabetic foot is largely related to differences in the standard care of the foot (Bus et al., 2020). The guidelines suggest that the best dressing selection should be based primarily on secretion control, comfort and cost to promote wound healing based on the usual optimal care in individuals with DFUs (Rayman et al., 2020). Dracaena Draconis (DD) has been used topically to treat foot ulcers. DD is also known as “Kirin Draconis”, which tastes sweet and salty. It has a flat nature, enters the liver and heart meridians, and has the following effects: promotes blood circulation and removes blood stasis, haemostasis, analgesia, astringent sores and regenerating muscles. It is extensively applied in traditional Chinese Medicine surgery to repair the wound. The Compendium of Materia Medica calls it “the holy medicine for activating blood circulation”. Its effectiveness has been highly praised by doctors in all dynasties and is an essential medicine for muscle regeneration. According to the summary of predecessors, Dracaena Draconis is the most frequently used drug in Shengji prescriptions recorded in ancient books of traditional Chinese medicine (Yanliang Yan, 2002). Dracorhodin perchlorate (DP, C17H15ClO7, 366.75 g/mol, Supplementary Figure S1) is the main extract of dracorhodin and currently used as substitute of Draconis in scientific research; it is widely used by researchers. Research pointed out that draconisin perchlorate preferentially activates EGFR on the surface of fibroblasts and then activates the phosphorylation of downstream pathways ERK and PI3K, resulting in the activation of a series of cell proliferation-related pathways. Thus, the regulation of cell proliferation is realised, promoting ulcer wound healing (Lin). This finding gives a theoretical foundation for the use of Dracaena Draconis to treat wound repair. The mechanisms and targets of DP in the treatment of DFU remain obscure. Similar to DP, potential treatments for DFU exist, such as paeoniflorin, mangiferin, and luteolin. Paeoniflorin effectively inhibits NLRP3 and NF-κB-mediated inflammation in DFU by inhibiting CXCR2 (Sun et al., 2021). Topical application of mangostin inhibits the prolongation of the inflammatory phase of diabetic wounds by suppressing inflammation and oxidative stress (Lwin et al., 2021). Luteolin promotes wound repair in DFU rats by inactivating NF-κB and upregulating Nrf2 to improve inflammation and oxidative stress (Chen et al., 2021).

DFU is associated with the variations of complicated metabolic physiologies. Meanwhile, as a helpful method, metabolomics can track the dynamic changes of pathological metabolites (Li et al., 2021). Nevertheless, traditional metabolomics is used to reflect the ultimate changes in disease and treatment (Fu C, 2019). Endogenous mechanisms underlying the changes of metabolites are unknown; these involve the producing process of these metabolites, their related proteins and pathways and which proteins DP acts on. As a result, metabolomics alone may limit DP’s application.

The concept of “network pharmacology” was first proposed by Hopkins. It is a method to analyse the potential therapeutic targets of drug intervention and disease (Deng et al., 2021). However, it is constrained by a single calculation method that relies on a publicly accessible database. Based on network pharmacology, it only predicts the possibilities of pathway analysis and compound-target combination (Zhong et al., 2018). Whether DP binds to target cells *in vivo* and whether DP has an inhibitory, activating, or ineffective combination effect on target cells are unknown. Metabolomics is a promising histological method for understanding biological mechanisms. Based on untargeted or targeted metabolomics analysis, metabolites in biological samples (tissues, cells, and others) were identified by comparing controls with altered groups, receiving different treatments, undergoing different levels of stress, dietary modifications or disease or condition-specific promotions using sophisticated analytical techniques, advanced data processing and statistical analysis (Canuto et al., 2017). Network pharmacology and metabolomics are bioinformatic approaches that enrich the analysis of pathways.

**FIGURE 1:**
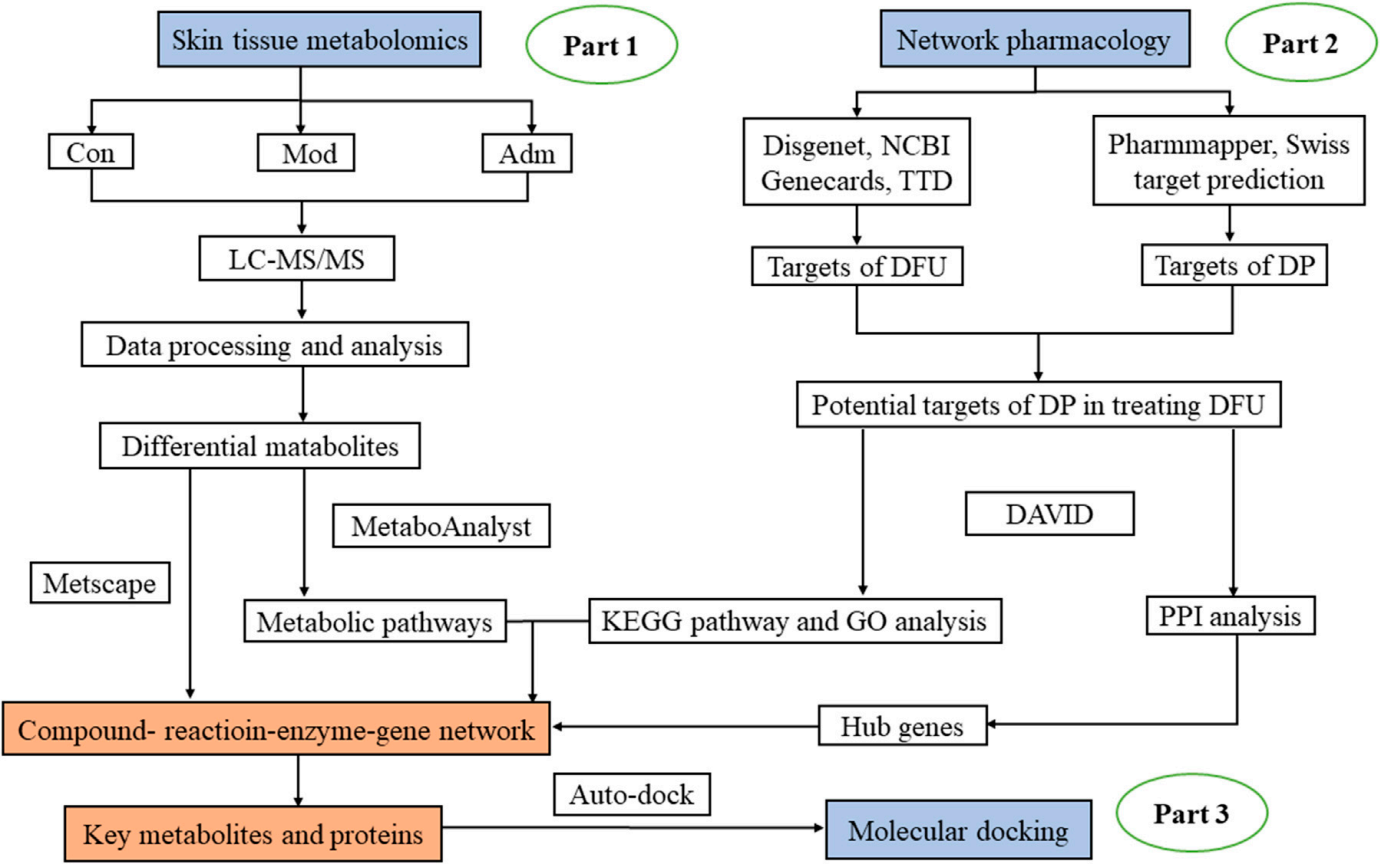
The flowchart of the integrated methods. The mechanisms of DP in treating DFU was explored with metabolomics of skin tissues (Part 1). Network pharmacology was performed to extract hub gene (Part 2). Through Part 1 and 2, important targets and metabolites were identified and connected. Molecular docking was conducted to further verify these core targets (Part 3).

Hence, we used a method based on combination of network pharmacology and metabolomics. The untargeted metabolomics was undertaken to explore DP’s effects on DFU and confirm the key metabolites. After that, with network pharmacology, we investigated the targets on which DP acted in the treatment of DFU and the targets and reactions that regulated the metabolites. The advantage of this strategy is as follows. On the one hand, short of validation with experiment of network pharmacology can be compensated; on the other hand, it addresses the shortness of targets’ binding to drug and molecular mechanisms in metabolomics. This method could help researchers better understand the therapeutic principles of natural compounds used to treat DFU. In this research, we applied for the first time a combined metabolomics and network pharmacology approach to explore the core targets and mechanisms of DP for DFU treatment. It sheds new light on the mechanistic study of DP in treating DFU. Figure 1 depicts the research flowchart.

## 2 Material and methods

### 2.1 Chemicals and kits

DP was purchased from Shanghai Yuanye (China). No: B21414-20 mg.

Jishuitan Hospital kindly donated L929 mouse fibroblasts cells. Hyclone provided Cell Counting Kit-8 (CCK-8), tryptone, Dulbecco’s modified eagle medium (DMEM), foetal bovine serum (FBS) and phosphate buffer saline (PBS, PH = 7.4) (United States). Methacrylate Gelatin (GelMA) was obtained from Suzhou Yongqinquan Intelligent Equipment Co., Ltd. Sigma-Aldrich (United States) provided streptozotocin (STZ), and citric acid/sodium citrate buffer was provided by Beijing Chengxin Kangrun Medical Equipment Co., Ltd. Ethacridine lactate solution (ELS) was from Hebei Wuluo Pharmaceutical Co., Ltd., Hebei, China. TNF-α, hs-CRP and IL-1 kits were from Quanzhou Ruixin Biotechnology Co., Ltd., Quanzhou, China. All chemicals in the experiment were analytical grade.

### 2.2 Cell proliferation experiment

The viability of L929 cells on a hydrogel/nanofiber dressing loaded with DP at various concentrations was assessed using the CCK-8 assay. The dressing was fixed in a 24-well plate, and 100 μL of L929 cells suspension (20000 cells/well) and 300 μL of cell culture medium were plated onto the sample and incubated at 37°C. The CCK-8 assay for testing cells viability on the dressing at each time interval (days 1, 3 and 5).

### 2.3 Experimental animals

Beijing University of Chinese Medicine Laboratory Animal Centre (Beijing, China) provided 7-week-old -specific-pathogen-free Sprague Dawley male rats weighing 200–250 g each (the production license number of animals was SCXK (jing) 2021-0006). All male rats were housed in a well-ventilated room with a temperature of 25°C and a 12-h dark-light cycle. They were given free access to water and food for free. The Animal care was carried out in accordance with the Beijing University of Chinese Medicine Laboratory Animal Centre Animal Care and Use Guidelines. Beijing University of Chinese Medicine approved the protocol of the Medical Ethics Committee under approval number 4-2021120302-4121.

### 2.4 Experimental animal design

#### 2.4.1 DM experimental model

After 40 SD rats were acclimatised to the basal diet for 1 week. Ten of them were randomly selected and fed with a normal diet to serve as the control group (Con). The remaining 30 rats were fed with high-fat and high-sugar diets for 1 month. Then all rats fasted for 12h, a diabetic rat model was prepared by the intraperitoneal injection of 40 mg/kg streptozotocin (STZ, STZ injection dose formula: body weight × STZ preset dosage)/1,000 = stz dosage (mg) solution. STZ is prepared into a solution with 1% concentration with citric acid/sodium citrate buffer before use, PH4.2 (Bonner-Weir S et al., 1983). Rats in the Con group were injected intraperitoneally with an equal volume of 0.1 mol/L citric acid/sodium citrate buffer without STZ. The diabetic rat was evaluated by drawing blood from the tail vein and measuring fasting blood glucose (FBG) 3 days after the injection of STZ with an Accu-Chek glucometer (Roche, Germany). The rats were observed to have polydipsia and polyuria, dirty back hair, wet padding and others. Having an FBG value of greater than 16.7 mmol/L was considered a sign of diabetes.

#### 2.4.2 DFU experimental model

Rats in the Con group and the diabetes model group were anaesthetized by intraperitoneal injection of 1% concentration of pentobarbital (40mg/kg) at a dose of 0.4ml/100g. The long hairs on the spine of the rats were removed with an electric razor. The modelling area was marked in the depilation area with a circular seal. Under sterile conditions, the skin in the modelling area was cut off to reach the fascia. A circular wound with a diameter of 1.5 cm was formed, and the animals were fed in a single cage. The wound was coated with glacial acetic acid once a day. A 5 cm *7 cm* 4-layer gauze block was used for binding and fixing. The diabetic skin ulcers were formed 1 week later. The success of the skin ulcer model was evaluated by the absence of granulation tissue growth around the wound, the appearance of swelling and abscesses in the skin and the absence of oozing blood with a large-headed needle prick (Jia et al., 2018).

FBG measurements were taken on days 0, 3, 7, 10, 14, 21, and 28 following the start of topical DP treatment on the wound. Wound area and wound healing rate were recorded on days 0, 3, 7, 10, 14, 21, and 28. The size of the wound area was measured using ImageJ, which is a java-based public image processing tool designed by the National Institutes of Health. Wound healing rate (%) was determined as follows (wound area before administration-unhealed wound area)/the wound area before administration) × 100%.

#### 2.4.3 Preparation of DP dressing (bilayer hydrogel/nanofiber dressing)

To prepare a homogeneous GelMA (20 w/v %) solution, 0.2 g GelMA was added to a PBS solution containing a photoinitiator and heated for 30 min at 60°C. Then, DP (3 μg) was added to the GelMA solution (1 ml) to form a homogeneous loaded-DP precursor solution. The electrospun mats were put in moulds to create the bilayer hydrogel/nanofiber dressing, and 400 μl of the loaded-DP hydrogel precursor solution (before UV crosslinking) was added to each mould. After that, UV crosslinking was done for 60 s. Eventually, DP dressing was made (Each DP dressing contains 1.2 ug DP).

#### 2.4.4 Grouping of experimental animals and methods of intervention

All male rats were assigned to four groups at random: Normal wound group (Con); Model group (Mod); Western medicine group (Wmg), in which the wound surface was externally applied with Ethacridine lactate solution (ELS) (Jia et al., 2018), wrapped and fixed with medical paper tape, once a day; and DP group, also known as Adm group. The topical application of DP dressing was performed in the Adm group. There was no need to change the DP dressing during the treatment. No intervention was applied to rats in the Con and Mod groups.

### 2.5 Blood sample and skin tissue collection

Blood samples were extracted from the abdominal aorta of the rat, placed in a 4°C refrigerator refrigerated for 30 min and centrifuged at 3,000 rpm for about 15 min for obtaining serum. The serum levels of TNF-α, IL-1 and hs-CRP were determined by using Enzyme-linked immunosorbent assay (ELISA). After the male rats were sacrificed, the wound skin tissue was removed and rapidly frozen in liquid nitrogen, transferred to a −80°C refrigerator, and subsequently used for the assays.

### 2.6 Pathological analysis

The dermal tissues around the wound of DFU rats on the 28th day were collected for pathological analysis to observe the microscopic changes during wound healing. The re-epithelialisation of damaged skin was estimated by hematoxylin-eosin (H&E)staining. To assess collagen formation and deposition, Masson staining was used.

Skin tissue fixed in 4% paraformaldehyde was subsequently cut into 1 cm diameter coin-sized tissue sections, which were then embedded in paraffin wax and transformed into pathological sections. Sections were dewaxed.

H&E staining: the dewaxed tissue sections were stained in hematoxylin staining solution for 3–5 min, washed in tap water, fractionated in fractionation solution, washed in tap water, returned to blue in return blue solution and rinsed in running water. Slices were dehydrated in 85% and 95% gradient alcohol for 5 min each and stained in eosin staining solution for 5 min. Slices are placed in anhydrous ethanol I for 5 min, anhydrous ethanol II for 5 min, anhydrous ethanol III for 5 min, dimethyl Ⅰ for 5 min and xylene Ⅱ for 5 min for transparency and sealed with neutral gum.

Masson staining: dewaxed tissue sections were soaked in potassium dichromate overnight and washed in tap water. Ferric hematoxylin A solution was mixed with B solution in equal proportions to form ferric hematoxylin staining solution. Slices were placed into ferric hematoxylin for 3 min, washed with tap water, differentiated with differentiation solution, washed with tap water, returned to blue with blue return solution and rinsed with running water. Slices were stained in Ponceau acid fuchsin staining buffer for 5–10 min and then rinsed with tap water. Phosphomolybdic acid in aqueous solution for 1–3 min After phosphomolybdic acid, without washing, directly into aniline blue staining solution for 3–6 min. The slices were divided with 1% glacial acetic acid and dehydrated in two tanks of anhydrous ethanol. The sections were placed in a third tank of anhydrous ethanol for 5 min, xylene for 5 min for transparency and neutral gum to seal the sections.

The H&E and Masson sections were then magnified and photographed using a light microscope (ZEISS ZEN3.4, Germany). Wound healing, collagen deposition and inflammation were examined using Image-Pro Plus 6.0 (Media Cybernetics, Inc, Rockville, MD, United States).

### 2.7 Cytokine assays

The TNF-α, hs-CRP and IL-1 were detected through ELISA. The experiments were carried out according to the manufacturer’s instructions. A microplate reader (Thermo Scientific Microplate Reader, United States) was used to measure the absorbance at 450 nm.

### 2.8 Skin tissue metabonomic analysis

#### 2.8.1 Metabolite extraction

Metabolite extraction (Dunn et al., 2011) was performed to control the quality of sample preparation. Tissue (25 mg) was weighed. The extraction reagent at 800 µl was added after precooling (methanol: acetonitrile: water (2:2:1, v/v/v) along with internal standard mixture 1 (IS1: L-Leucine-d3, L-PHENYLALANINE) and 2 (IS2: L-Tryptophan-d5, Progesterone-2,3,4-13C3). Then, metabolites were extracted. After homogenising the samples for 5 min with a TissueLyser (JXFSTPRP, China), they were sonicated for 10 min and incubated for 1 h at −20°C. After centrifuging the samples at 25,000 rpm and 4°C for 15 min, the transferred supernatant was subjected to vacuum freeze drying. We resuspended the metabolites in 200 µl of 10% methanol and then sonicated for 10 min at 4°C before centrifugation for 15 min at 25,000 rpm. The supernatants were transferred to autosampler vials for LC-MS analysis. For evaluating the reproducibility of the entire Liquid Chromatography-Mass Spectrometry (LC-MS) analysis, quality control (QC) was prepared by pooling the same volume of all samples.

#### 2.8.2 LC-MS/MS analysis

Using a Waters 2D UPLC (Waters, United States) tandem Q Exactive high resolution mass spectrometer (Thermo Fisher Scientific, United States), metabolite was separated and detected. A Waters 2D UPLC (Waters, United States) coupled to a Thermo Fisher Scientific Q-Exactive mass spectrometer with a heated electrospray ionisation (HESI) source and controlled by the Xcalibur 2.3 software program (Thermo Fisher Scientific, Waltham, MA, United States) was used for sample analysis. Waters ACQUITY UPLC BEH C_18_ column (1.7 µm, 2.1 mm × 100 mm, Waters, United States) was used to perform chromatographic separation. We set the temperature of a column to 45°C. There were 0.1% formic acid (A) and acetonitrile (B) in the mobile phase in the positive mode and 10 mM ammonium formate (A) and acetonitrile (B) in the negative mode. The gradient conditions are shown in Supplementary Table S1. The flow rate was 0.35 ml/min, and the injection volume was 5 μl. We injected one QC sample for every 10 samples.

#### 2.8.3 Data processing and analysis

Using Compound Discoverer 3.1 (Thermo Fisher Scientific, United States), the LC-MS/MS raw data (raw file) was processed, including retention time correction, peak extraction, additive ion pooling, background peak labelling, missing value filling and metabolite identification, before information on retention time, molecular weight and peak area was exported. The identification of metabolites was the result of a combination of mzCloud, Human Metabolome Database (HMDB), Kyoto Encyclopedia of Genes and Genomes (KEGG) and LipidMaps. Fragment Mass Tolerance lower than 10 ppm, Precursor Mass Tolerance lower than ppm and Tolerance for RT lower than 0.2 min are the main parameters of metabolite identification.

We imported result data into metaX for preprocessing to do the following: 1. To obtain relative peak area, normalise the data using Probabilistic Quotient Normalization [PQN (Di Guida et al., 2016)]; 2. To use QC-RLSC (quality control-based robust LOESS signal correction) to correct the batch effect; and 3. To determine the relative peak area’s CV (Coefficient of Variation) in each QC sample, and remove compounds with a CV of more than 30%.

After standardisation, we screened different metabolites based on VIP>1 and *p* < 0.05 with principal component analysis (PCA), partial least-squares discrimination analysis (PLS-DA), and T-test. Using the HMDB database, the names of each metabolite were standardised. Using the PeakView software, potential endogenous metabolites were identified. It was possible to predict the metabolic pathways connected to DFU by using MetaboAnalyst 5.0 (https://www.metaboanalyst.ca/).

### 2.9 The construction of network pharmacology

With cytoscape 3.8.2, the network was constructed to visualise the the relationship of metabolite-protein-pathway and to elaborate the hub metabolites and related proteins. The steps were as follows (Figure 1). 1) The targets of DFU were obtained *via* searching the keywords “diabetic foot ulcer” in the gene maps of Disgenet (https://www.disgenet.org/), NCBI (https://www.ncbi.nlm.nih.gov/), Therapeutic Target Database (TTD, http://db.idrblab.net/ttd/) and genecards (https://www.gene-cards.org/). 2) The molecular targets of DP were filtered by searching Pharmmapper (lilab-ecust.cn/Pharmmapper/index.chromatographic separation.html) and SwissTargetPrediction (http://www.swisstargetprediction.ch/) for the keywords “Dracorhodin Perchlorate”. To screen the related proteins in *Rattus norvegicus*, the structure of DP was imported into SwissTargetPrediction and Pharmmapper, respectively. 3) The intersection of 1) and 2) was thought to be a potential target of DP in the treatment of DFU. With UniProtKB (http://www.uniprot.org/), the names of targets were standardised. 4) Targets were imported into STRING 11.0 (https://string-db.org/) and protein-protein interaction (PPI) network was constructed using Cytoscape 3.8.2. 5) Through the DAVID (https://david.ncifcrf.gov/) database, the pathway and Gene Ontology (GO) enrichments of potential targets were analysed. We set the *p* < 0.05 for the standard of the KEGG pathway. 6) To achieve the compound-reaction-enzyme-gene network, we imported the identified differential metabolites in metabolomics and the intersection targets of DP in treating DFU into MetScape of Cytoscape. Then, the crucial metabolites and proteins were recognised.

### 2.10 Molecular docking

PubChem (https://www.ncbi.nlm.nih.gov/pccompound/?term) was used to obtain the 3D structure of DP (PubChem CID: 6443665). With RCSB Protein Data Bank (https://www.rcsb.org/), we downloaded the structures of targets. Seven targets were investigated: Glutathione S-Transferase Mu 1 (GSTM1, PDB ID:1YJ6), Thymidylate Synthetase (TYMS, PDB ID:1RTS) (Rogerio R. Sotelo-Mundo, 1999), and Phenylalanine Hydroxylase (PAH, PDB ID: 3PAH) (Heidi Erlandsen, 1998), and Dopa Decarboxylase (DDC, PDB ID: 3RCH) (Giardina et al., 2011), Dihydrofolate Reductase (DHFR, PDB ID:4M6J) (Bhabha et al., 2013), Catalase (CAT, PDB ID:1dgb) and Phosphoglucomutase 1 (PGM1, PDB ID: 6uiq) (Stiers and Beamer, 2018). AutoDockTools 1.5.6 was used to convert DP (mol2 format) and targets (pdb format) to pdbqt format (Deng et al., 2021). Molecular docking was then conducted with Autodock Vina. Supplementary Table S2 lists coordinates of active pockets in targets. Discovery Studio 2019 was used to visualise the docking results.

### 2.11 Statistical analysis

Presented data as mean±standard deviation (mean±SD). With one-way ANOVA, statistical analysis was performed. GraphPad Prism (Inc, La Jolla, CA, United States) 9.1 was used for Dunnett’s multiple comparisons. *p* < 0.05 was defined as the significance. T-tests were conducted on skin metabolome data.

## 3 Results

### 3.1 Results of cell proliferation experiment

**FIGURE 2:**
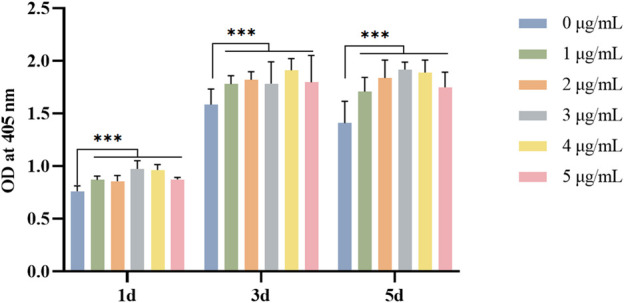
Effect of DP with different concentration on fibroblast viability in different days. *p<0.05, **p<0.01 and ***p<0.001.

The L929 cells showed a trend of increment on GelMA hydrogels loaded with Draconis perchlorate. The results of the cell proliferation experiment revealed that DP could promote the proliferation and vitality of fibroblasts and had the potential to promote DFU wound healing. We found the optimal dose of DP to be 3 μg (as shown in Figure 2).

### 3.2 Animal experiment results

#### 3.2.1 General conditions of rats

Thirty rats on a high-fat and high-sugar diet were injected with 40 mg/kg streptozotocin (STZ) solution intraperitoneally, and the blood glucose was continuously measured for 3 days. Finally, 18 diabetes rats with blood glucose >16.7 mmol/L were included for the modelling of the DFU model. In the Con group, six rats were made normal wound models. During the modelling period, rats that failed or died were excluded. Finally, 24 rats were included, six each in the Con, Mod, Wmg and Adm groups. Compared with the Con, all diabetes rats showed poor mental state, withered and yellow hair, obvious depilation, significantly reduced activity and other symptoms. At the same time, they could drink more, eat more, urinate more and lose weight obviously. The longer the time was, the more obvious the symptoms were.

#### 3.2.2 Topical application of DP on FBG levels in DFU rats

**FIGURE 3:**
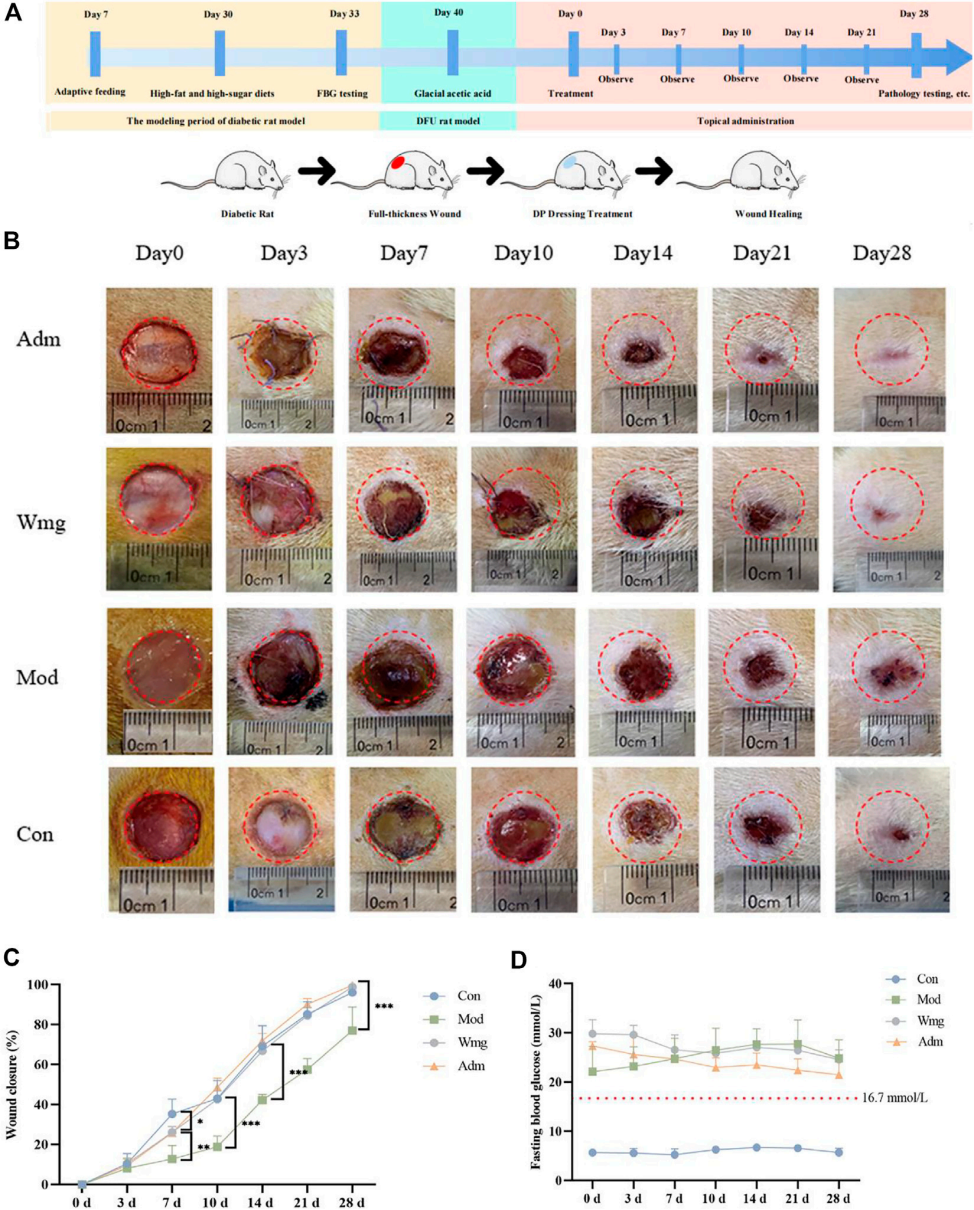
DP improves the wounds of DFU rats. **(A)** The time diagram of the experiment. **(B)** Representative figures of different time points of healing area of DFU rats. **(C)** Wound closure (%) of different groups at days 0, 3, 7, 14, 21 and 28. *p < 0.05 represent significance. **(D)** Images present the levels of FBG on 0th, 3th, 7th, 10th, 14th, 21st and 28th day.

The level of FBG remained stable in all experimental groups throughout the treatment period, i.e., on days 0, 7, 14, 21, and 28, respectively (Figure 3D). This finding showed that the DFU rat model in this study was successful. The blood glucose concentration of the Adm group was lower than that of the Mod group, suggesting that topical application of DP had a certain hypoglycaemic effect.

#### 3.2.3 Macroscopic effect of DP treating wound

Figure 3A depicts the timing diagram of the experiment, divided into three periods, the diabetic rat modeling phase, the DFU rat modeling phase, and the DP dressing administration phase. Figure 3B depicted the closure area of wound after the topical application of DP from days 0–28. The DP group showed more enhanced and faster wound closure than the Wmg and Mod groups. In terms of wound healing rates, on the seventh day, the wound healing rates were higher in the Adm group than those in the Mod and Wmg groups, as shown in Figure 3C. However, the wound healing rates of the Con group were great on the seventh day. Possibly, the metabolic disorder and inflammatory state in DFU rats influenced the wound closure. On the 14th day, a lower rate of wound healing was found in the Wmg group compared with the Adm group. On the 28th day, 99.6% of the Adm group’s wounds were closed. The rates of wound closure were 98.8% and 77% in the Wmg and Mod groups. Macroscopic analysis showed that the DP accelerated healing more quickly than the Wmg treatment.

Compared with the Con group, the Mod had poorer wound healing by day 28, and this finding was associated with high blood glucose and inflammation in the Mod. These inhibited the wound healing rate. When compared with the Mod group, the Adm group wound healed better. Compared with the Wmg group, the Adm group showed better results. The study’s findings revealed that the wound healing rate of the Adm group was greater than that in the Wmg group and greater than that in the Mod group.

#### 3.2.4 H&E and masson staining

**FIGURE 4:**
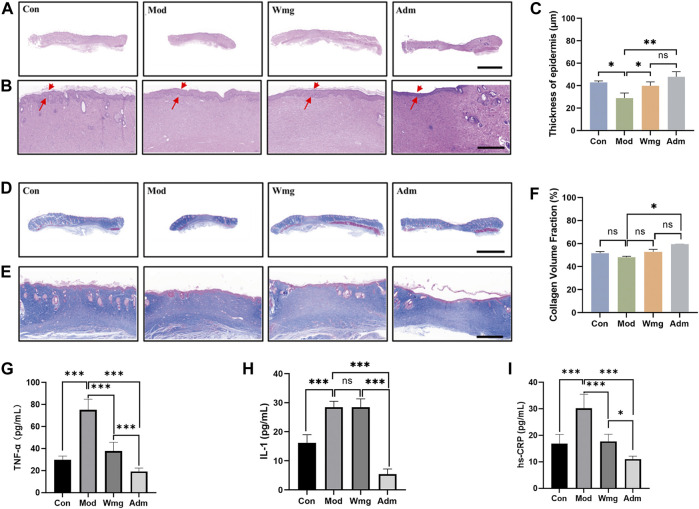
H&E staining, Masson staining and cytokine assay of different groups of wound tissues. **(A–B)** Representative images of wound sections with H&E staining on day 28 (scale bar = 5 mm, scale bar = 500 μm). Red arrow indicates regenerating epidermis. **(C)** Thickness of epidermis (*p < 0.05, **p < 0.01 and ***p < 0.001). **(D–E)** Representative images of wound sections with Masson’s trichrome staining on day 28 (Masson’s trichrome: scale bar = 5 mm, scale bar = 1,000 μm). The blue colored ones are collagen fibres and collagen bundles, and the red ones are muscle fibres. **(F)** Fraction of collagen volume in wound tissue (*p < 0.05, **p < 0.01 and ***p < 0.001). **(G)** The level of TNF-α of in each group. **(H)** The level of IL-1 of in each group. **(I)** The level of hs-CRP of in each group.

Chronic wound healing is a complex process that includes wound contraction, granulation formation, re-epithelialisation and collagen deposition. As a result, histological analysis was performed to assess the quality of regenerated epidermis in the defect. The re-epithelialisation of damaged skin was estimated by H&E staining. H&E staining revealed that a new epidermis formed in the damaged area, and the dermis was repaired by large amounts of connective tissue. Compared with the Mod group, the Adm group had a complete epidermis covering the wounds and more apparent granulation tissue on the 28th day after the application of DP. The tissue was closer to the structure of normal skin (Figures 4A,B). At the same time, the quantitative statistics of the thickness of regenerated epidermis showed that the thickness of the new epidermis in the Adm group was 47.5 μm, which was obviously greater than that in other groups (Figure 4C). The Adm group would show an effectively enhanced re-epithelialisation process compared with other groups.

The abundant deposition of collagen fibres in the dermis can strengthen newly formed skin tissue and constitute a suitable microenvironment for wound closure. To assess collagen formation and deposition, Masson staining was used. In the Adm group, the dermis had large deposits of collagen fibres and good collagen bundles, whereas, in the Mod group, the wounds had only small deposits of collagen fibres and poorly developed collagen bundles (Figures 4D,E). Quantitative analysis also revealed that the proportions of collagen volume in the dermis were 51.7%, 48.1%, 52.8%, and 59.5% in Con, Mod, Wmg and Adm, respectively (Figure 4F). Therefore, the Adm group healed the fastest wounds and promoted collagen deposition, thereby accelerating wound healing.

#### 3.2.5 Elisa analysis

This study found that the Adm group’s TNF-α level was lower than that in the Mod group, and the Adm group’s TNF-α content was only 0.255 times that of the Mod group and 0.5 times that of the Wmg group, which was similar to that of the Con group (Figure 4G). The IL-1 content of the Adm group was lower than that in Mod and Wmg groups in Figure 4H. Similarly, as shown in Figure 4I, the hs-CRP content of the Adm group was lower than those in Mod and Wmg groups. DP could effectively alleviate the inflammatory response of DFU rat wounds, regulate macrophage transformation from pro-inflammatory M1 to repair M2, accelerate the transformation from inflammation to proliferation and maintain stable wound remodelling.

### 3.3 Metabolomics profiling

Following data preprocessing, a total of 1983 features in skin tissue were determined (Supplementary Material S1). By using Quality Control (QC) samples, the stability and repeatability of metabolomics were assessed. Supplementary Figure S2 depicts that the QC samples were within the range of ± 2std, regardless of the positive and negative ion modes, suggesting that the equipment was stable and reliable, and the method was reproducible. There were large peak capacities and good peak shapes in the sample, as can be seen from the base peak chromatogram (BPC) char (Supplementary Figure S3).

**FIGURE 5:**
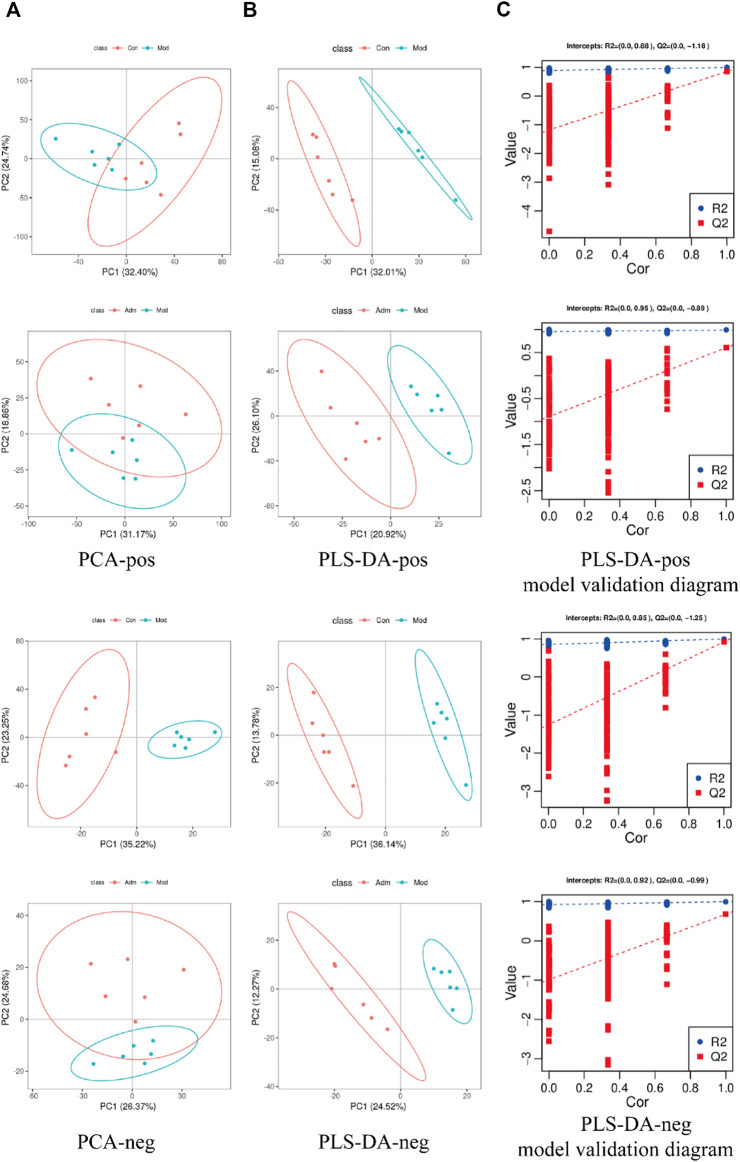
Score chart of PCA, PLS-DA model and PLS-DA model response permutation testing (RPT). **(A)** Score chart of PCA analysis model pos and neg **(B)**. Score chart of PLS-DA analysis model pos and neg. **(C)** PLS-DA model validation diagram.

We used the PCA and PLS-DA to examine the separation among the Adm, Mod and Con groups. From the PCA of positive (pos) and negative (neg) models, the samples of different groups have been separated (Figure 5A). PLS-DA disclosed that the same group clustered together, whereas different groups distinguished well (Figure 5B). The PLS-DA parameters of skin tissue samples were R2>0, Q2<0 (Validation parameters) (Supplementary Table S3), which indicated that the model is not over-fitted and is relatively stable.

### 3.4 Metabolites identification and pathway analysis

PLS-DA was performed to identify the differently expressed metabolites. There was good separation in each PLS-DA model, suggesting that sample classification information had a good explanatory ability. Additionally, permutation test demonstrated that the models were trustworthy and non-overfitting (Figure 5C), which consistent with the result of Supplementary Table S3.

**FIGURE 6:**
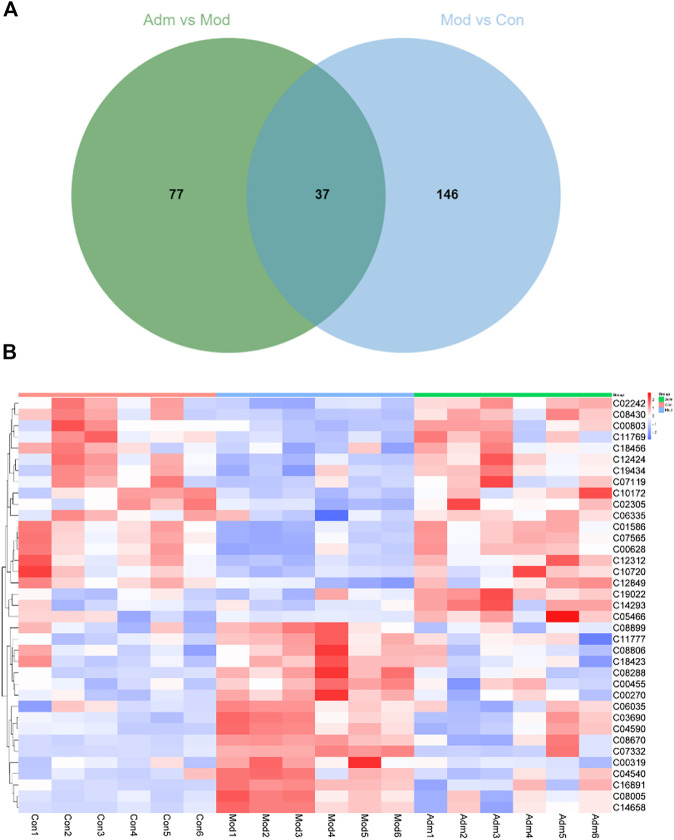
The differential metabolites in DFU rats treated with DP. **(A)** Venn diagrams of the common metabolites related to DFU and DP treatment. **(B)** The heat map of potential metabolites.

According to *p* < 0.05 and VIP >1, 183 differentially expressed metabolites were identified between the Con and Mod groups (Supplementary Material S2). There were 114 metabolites that differed in expression between the Adm and Mod groups (Supplementary Material S3). The two groups were intersected (http://jvenn.toulouse.inra.fr/app/example.html), and then, 37 differential metabolites of the effect of DP on DFU are presented (Figure 6A).

We established heat maps to depict the distinctions in metabolites between the three groups. According to Figure 6B, all candidate metabolites were altered in the Mod group, and the majority were reversed in the Adm group, reflecting that DP treatment could improve metabolic disorder.

**FIGURE 7:**
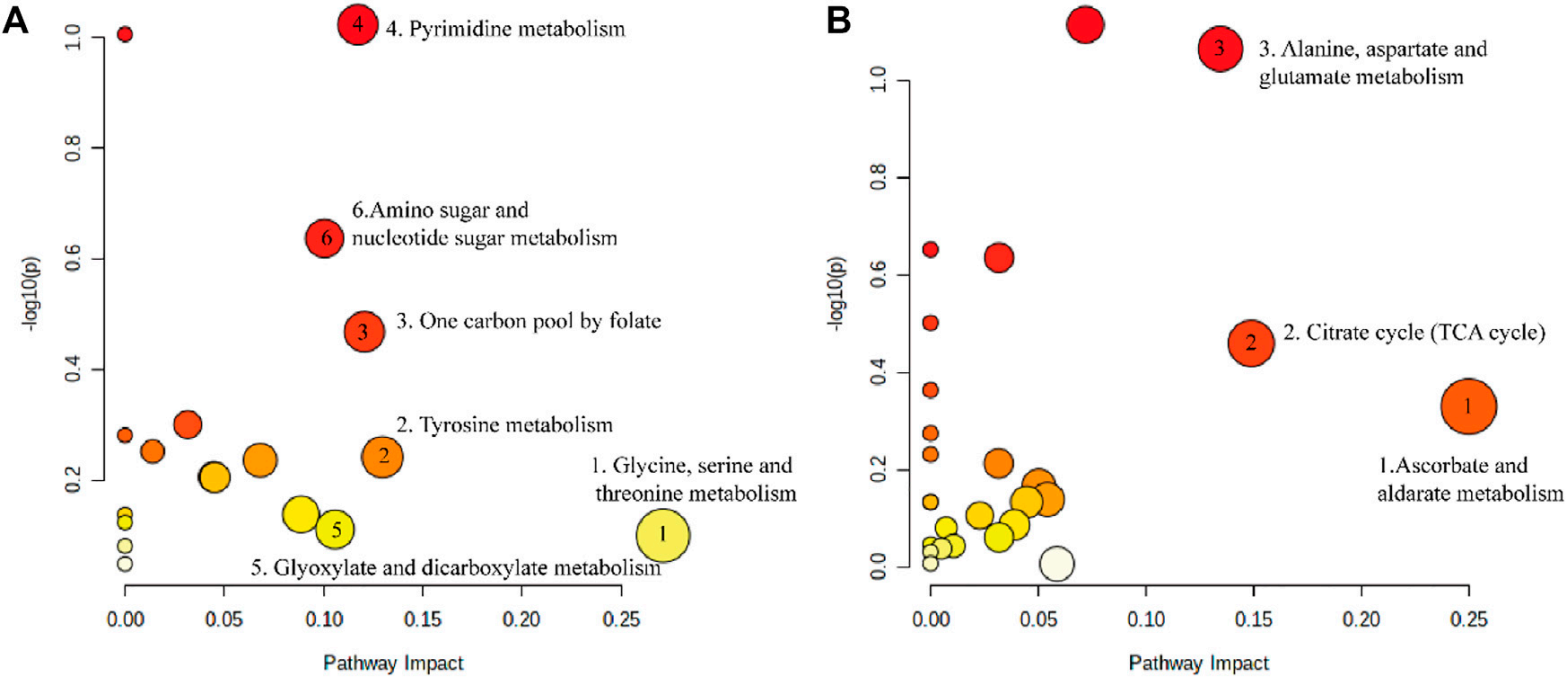
The metabolic pathways enriched by significant metabolite. **(A)** Metabolic pathway of DP in the treatment of DFU. **(B)** Metabolic pathway disorder caused by DFU.

We imported 114 different metabolites into MetaboAnalyst 5.0 to investigate the metabolic pathways of DP in the treatment of DFU rats. As shown in Figure 7A, six pathways were screened based on pathway impact> 0.1, including the following: Glycine, serine and threonine metabolism; Tyrosine metabolism; One carbon pool by folate; Pyrimidine metabolism; Glyoxylate and dicarboxylate metabolism; Amino sugar and nucleotide sugar metabolism. The metabolites related to these pathways were as follows: Glycine; Dopamine; 2,5-Dihydroxybenzoate; 5,10-Methenyltetrahydrofolate; Uridine; Cytidine 5′-monophosphate (CMP); Thymidine; Thymine; N-Acetylneuraminate; N-Acetyl-alpha-D-glucosamine 1-phosphate; and D-Glucosamine 6-phosphate. Tyrosine metabolism is most relevant to the effects on skin tissues. As shown in Figure 7B, three pathways had an impact of >0.1, including the following: ascorbate and aldarate metabolism; citrate cycle (TCA cycle); and alanine, aspartate and glutamate metabolism.

**FIGURE 8:**
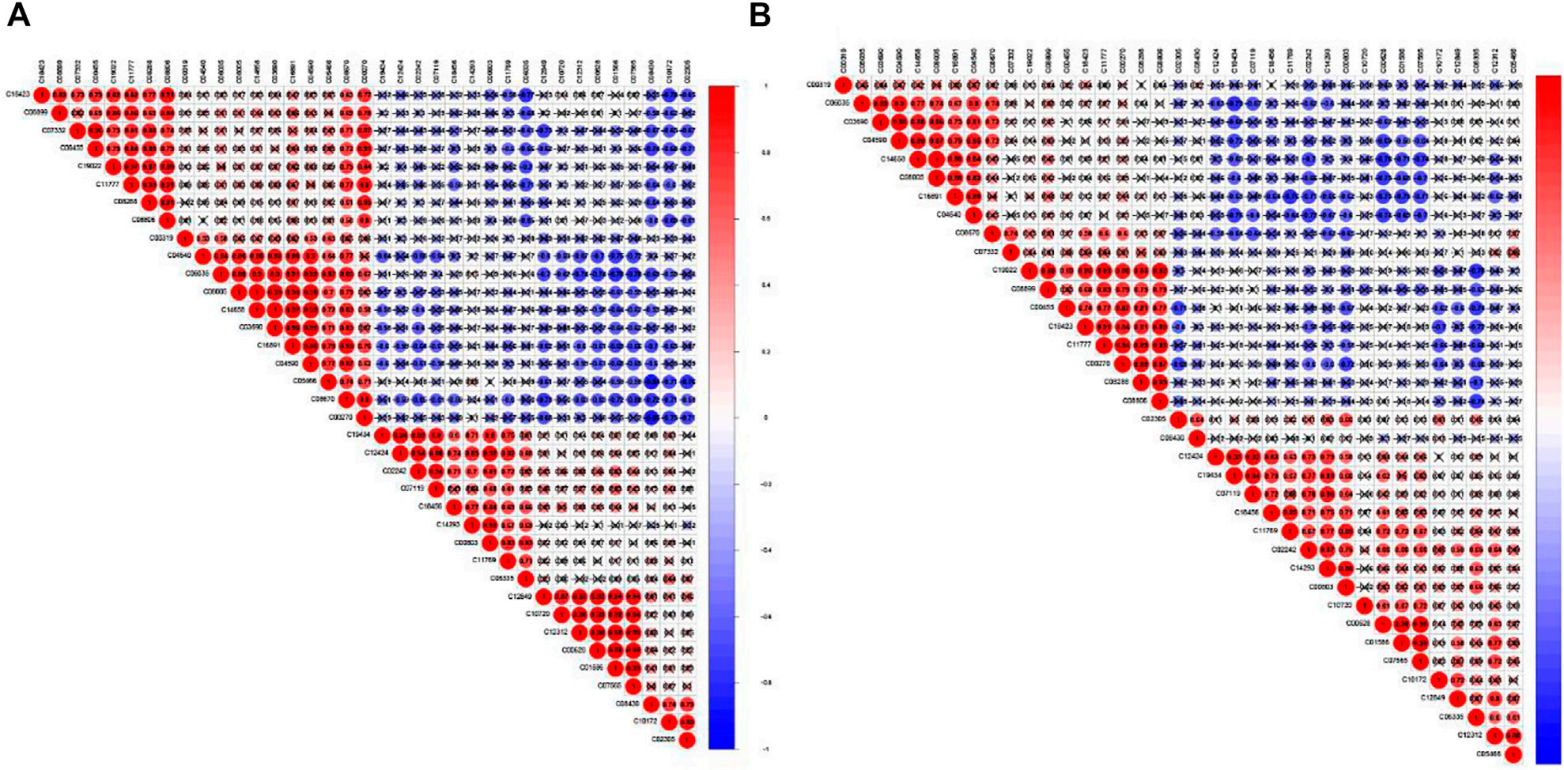
Correlation matrix of interaction in 37 differential metabolites. Coefficients of negative correlation (blue) and positive correlation (red) were plotted. The closer that the correlation coefficient was to 1, the redder the colour was, and the greater the positive correlation was. The closer that the correlation coefficient was to -1, the bluer the colour and the greater the negative correlation were. **(A)** The correlation heatmap of Adm and Mod groups. **(B)** The correlation heatmap of Mod and Con groups.

Furthermore, we made a correlation heatmap by “corrplot” package in R language to explore the correlation of different metabolites. As shown in Figure 8A, Pearson correlation analysis demonstrated that the C03690 and C04590, C00628 and C01586 and C07565, C01586 and C07565 were strongly correlated, and their correlation coefficients were 0.98, 0.96, 0.98, and 0.99, respectively. Figure 8B shows that the highest correlation was observed for C08005 and C1465 with a correlation coefficient of 1. C08005 and C03690 and C16891 and C04590 were also strongly correlated, and their correlation coefficients were 0.99. The names of 37 metabolites are presented in Table 1 and Supplementary Material S4.

**TABLE 1:** The differential metabolites in DP-treated DFU rats.

Rank	Metabolites	Formula	Molecular weight	RT (min)	MS/MS	Keggid	Adm vs. mod		Mod vs. con	
							VIP	*p*-value	VIP	*p*-value
1	8-hydroxyquinoline	C9 H7 N O	145.053	2.358	0.0003; 1.8363	C19434	2.3506	0.0084	1.386	0.0478
2	Binapacryl	C15 H18 N2 O6	322.1165	3.374	0;-0.006	C19022	2.1745	0.0147	1.8152	0.003
3	Cyproconazole	C15 H18 Cl N3 O	291.1141	5.322	0.0002; 0.7606	C18456	1.6722	0.0261	1.5941	0.0227
4	Thiodicarb	C10 H18 N4 O4 S3	354.0498	0.62	0.0008; 2.1597	C18423	1.3454	0.024	1.0393	0.0181
5	Callystatin a	C29 H44 O4	456.3221	9.366	-0.0019; -4.1474	C16891	1.7808	0.0207	3.4022	0
6	2062850	C23 H34 O3	358.2508	7.277	0;-0.0539	C14658	2.3474	0.0208	3.1382	0
7	Furaspor	C6 H7 N O4	157.0378	4.361	0.0003; 1.9149	C14293	1.7549	0.018	1.5618	0.007
8	Aklavinone	C22 H20 O8	412.1164	2.754	0.0006; 1.5147	C12424	2.8943	0.0014	1.6729	0.0096
9	2-oxindole	C8 H7 N O	133.053	4.945	0.0002; 1.7935	C12312	2.0915	0.0027	1.5062	0.006
10	Iproniazid	C9 H13 N3 O	179.1062	3.528	0.0003; 1.9039	C11777	1.9009	0.044	1.3339	0.0018
11	R-soterenol	C12 H20 N2 O4 S	288.1144	4	0;-0.0067	C11769	1.3351	0.0316	1.733	0.0071
12	Stachydrine	C7 H13 N O2	143.0947	0.698	0.0001; 0.6792	C10172	2.0737	0.0096	1.1946	0.0082
13	Gitogenin	C27 H44 O4	432.324	9.74	0.0001; 0.1832	C08899	1.7512	0.0046	1.2122	0.035
14	(+)-cassaine	C24 H39 N O4	405.288	6.327	0.0001; 0.1614	C08670	1.4728	0.0453	3.5603	0
15	Indospicine	C7 H15 N3 O2	173.1168	2.965	0.0004; 2.1138	C08288	3.0717	0.0007	2.6712	0.0003
16	Alfentanil	C21 H32 N6 O3	416.2541	7.273	0.0005; 1.1703	C08005	2.3074	0.0204	2.7639	0.0003
17	Benzyl succinate	C18 H18 O4	298.1218	4.975	0.0013; 4.4335	C07332	3.9133	0.0107	3.6341	0
18	4-methylene-2-oxoglutarate	C6 H6 O5	158.0218	4.403	0.0003; 1.7232	C06035	1.1701	0.0467	2.0472	0.0089
19	Glycochenodeoxycholate	C26 H43 N O5	449.3143	7.697	0.0001; 0.3032	C05466	2.2593	0.0175	1.0836	0.028
20	Bis(2-ethylhexyl) phthalate	C24 H38 O4	390.2771	7.834	0.0001; 0.3222	C03690	1.5677	0.0487	3.3478	0
21	Phosphocreatine	C4 H10 N3 O5 P	211.0361	0.741	0.0003; 1.2875	C02305	2.1276	0.0059	1.1389	0.0159
22	Epiguanine	C6 H7 N5 O	165.0654	1.028	0.0003; 1.8545	C02242	2.163	0.0001	1.0414	0.0178
23	Hippurate	C9 H9 N O3	179.0586	3.845	0.0003; 1.7581	C01586	2.6146	0.0003	2.0338	0.0005
24	Β-nicotinamide mononucleotide	C11 H15 N2 O8 P	334.0566	1.221	0; 0.0514	C00455	1.5921	0.0426	1.5837	0.0008
25	D-sphingosine	C18 H37 N O2	299.2824	8.271	-0.0001;-0.2512	C00319	1.4659	0.0068	1.064	0.0127
26	4-phenolsulfonic acid	C6 H6 O4 S	173.9986	2.93	0;-0.2785	C12849	1.6561	0.0196	1.8013	0.0007
27	90,049	C14 H18 O7	298.1048	4.532	-0.0004;-1.3638	C10720	1.6011	0.0198	1.1469	0.0209
28	Cucurbitacin s	C30 H42 O6	498.3005	9.827	0.0024; 4.7174	C08806	1.4406	0.0256	1.4663	0.0261
29	Convicine	C10 H15 N3 O8	305.0854	2.787	-0.0005;-1.5802	C08430	2.1701	0.0011	1.1435	0.0231
30	Acetanilide	C8 H9 N O	135.0684	2.955	0; 0.148	C07565	2.4849	0.0004	1.8611	0.0007
31	Medroxyprogesterone	C22 H32 O3	344.2347	8.933	-0.0004;-1.2028	C07119	1.2394	0.0448	1.3328	0.0188
32	Sulfanilic acid	C6 H7 N O3 S	173.0146	4.528	0;-0.0987	C06335	1.5748	0.0436	1.3003	0.041
33	Sn-3-o-(geranylgeranyl)glycerol 1-phosphate	C23 H41 O6 P	444.2638	7.595	-0.0002;-0.5344	C04590	1.4605	0.0266	3.3553	0
34	N4-(beta-n-acetyl-d-glucosaminyl)-l-asparagine	C12 H21 N3 O8	335.1324	0.695	-0.0004;-1.2919	C04540	1.1283	0.016	1.1051	0.0034
35	Valeric acid	C5 H10 O2	102.0681	3.456	0; 0.0327	C00803	1.6896	0.0078	1.2316	0.0283
36	Gentisic acid	C7 H6 O4	154.0266	3.552	0;-0.1854	C00628	2.2384	0.0024	1.9753	0.0015
37	N-acetylneuraminate	C11 H19 N O9	309.1055	0.696	-0.0005;-1.5163	C00270	1.0222	0.0063	1.6635	0.0001

### 3.5 Result of network pharmacology

For further investigating the mechanisms of DP in treating DFU, we conducted network pharmacology. First, the targets of DFU through Disgenet, NCBI, TTD and Genecards and targets of DP from Pharmmapper and SwissTargetPrediction were gathered. After the intersection of DP and DFU-related targets, we identified 108 potential targets for DP-treating DFU (Supplementary Figure S4). With the UniProt database, we normalised common targets to the official symbols.

**FIGURE 9:**
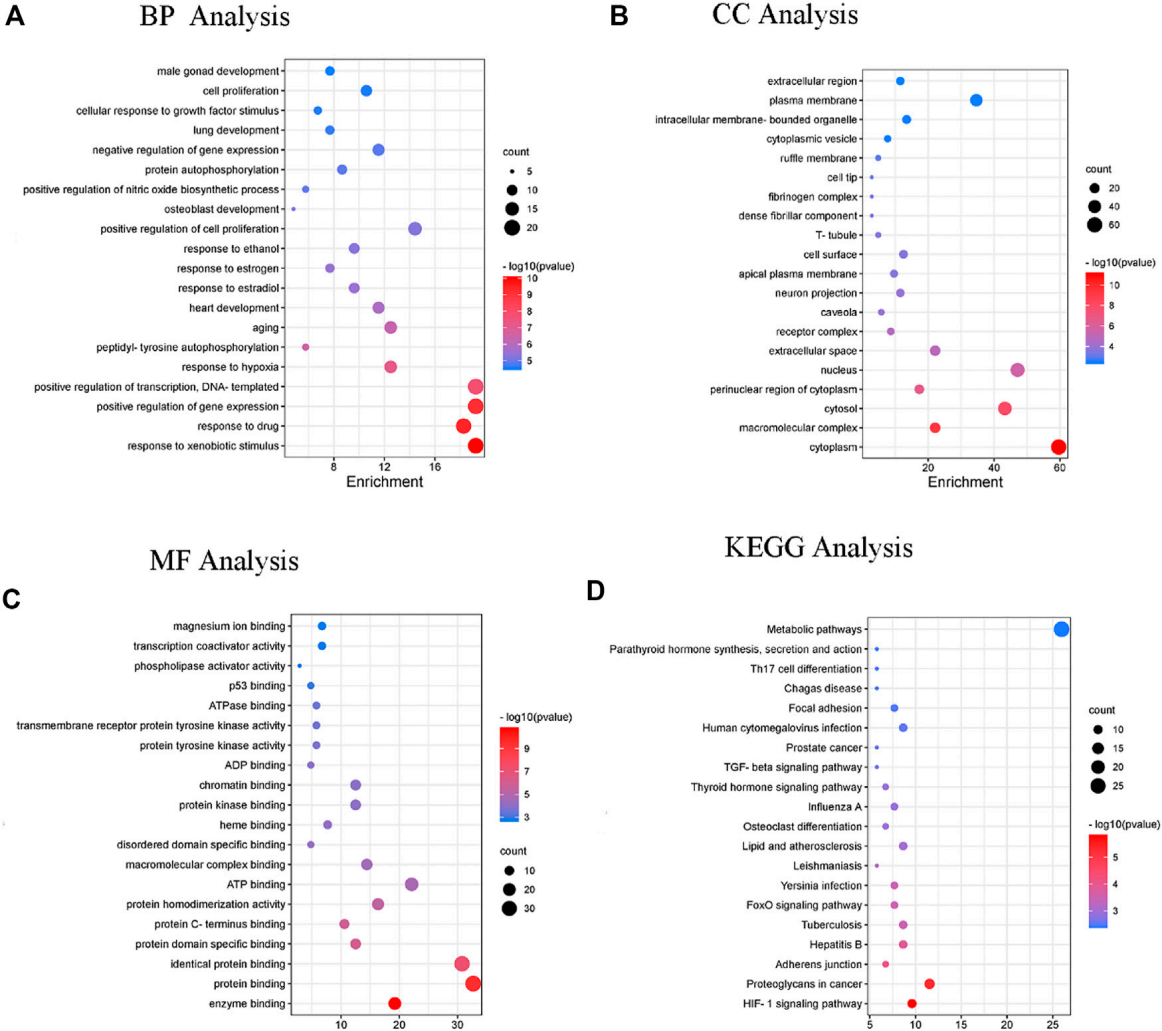
The enrichment analysis of GO and KEGG pathway of DP for the treatment of DFU. Enriched GO terms are for **(A)** BP analysis **(B)** CC analysis **(C)** MF analysis; and **(D)** KEGG pathway analysis.

Subsequently, DAVID was used for gene ontology and pathway analysis. The findings revealed that the biological process (BP) of DP in the treatment of DFU evolved primarily in response to drugs, cellular response to growth factor stimulus, hypoxia and positive regulation of cell proliferation. The cellular component (CC) was primarily involved in the cytoplasm, macromolecular complex, cytosol and perinuclear region of the cytoplasm. The molecular function (MF) was principally engaged in enzyme, protein and macromolecular complex binding. The KEGG enrichment analysis revealed that the HIF-1 signaling, TGF-beta signaling and metabolic pathways were the pathways most significantly impacted (Figure 9.)

We constituted a PPI network through Cytoscape to pinpoint key genes of DP in the treatment of DFU. An overview of the relationships between 108 targets was presented in Supplementary Figure S5. The top seven genes were chosen as hub genes (TGFBR1, FNIFNG, Src, FN1, Hsp90aa1, Cat and Notch1).

### 3.6 Integration of network pharmacology and metabolomics

**FIGURE 10:**
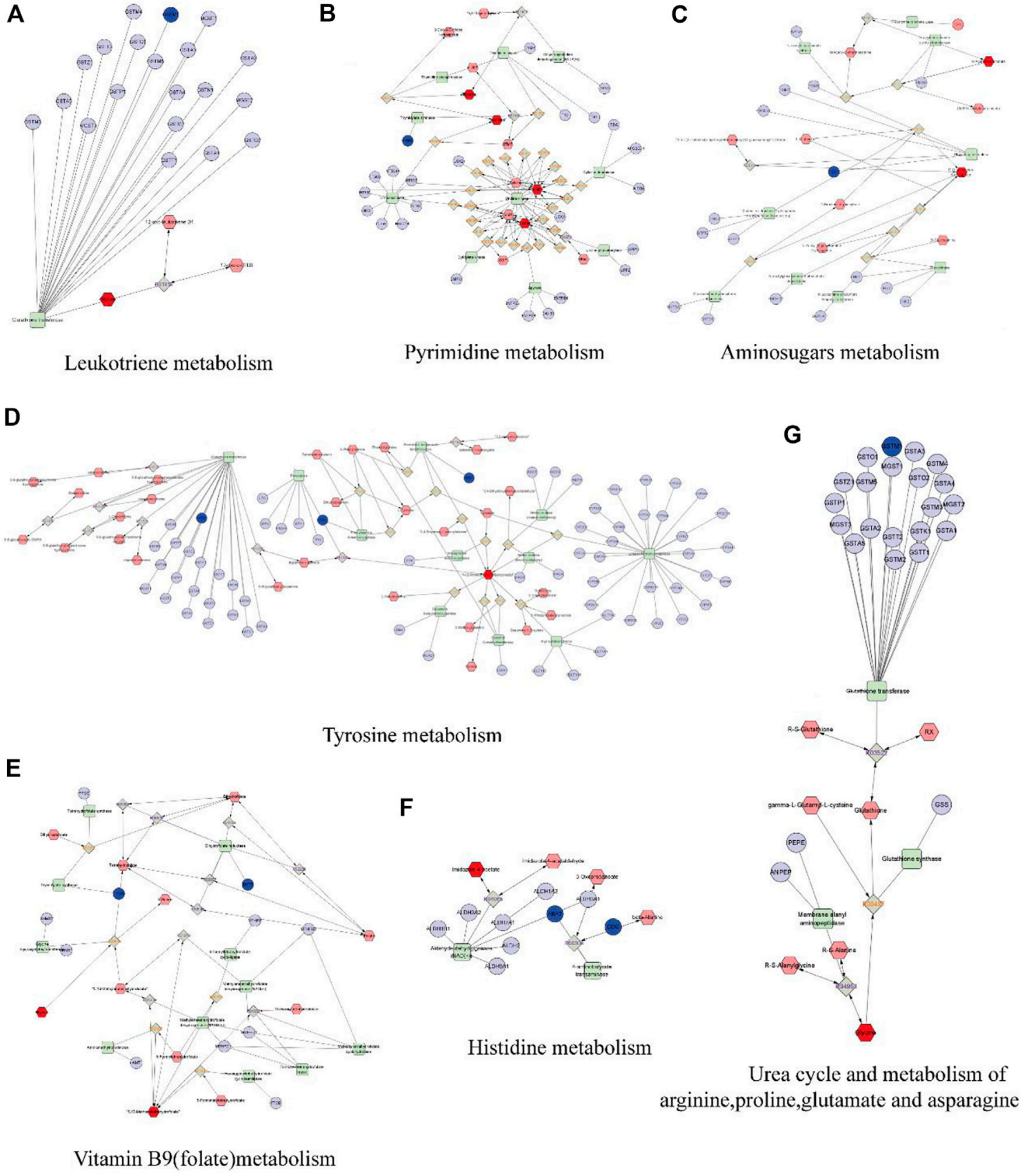
The compound-reaction-enzyme-gene networks of the crucial targets and metabolites. The red hexagons represent the active compounds. The grey diamonds represent the reaction. The green round rectangle represent enzyme. The light purple circles present general genes, and the blue circles represent key genes. **(A)** Leukotriene metabolism. **(B)** Pyrimidine metabolism. **(C)** Amino sugars metabolism. **(D)** Tyrosine metabolism. **(E)** Vitamin B9 (folate) metabolism. **(F)** Histidine metabolism. **(G)** Urea cycle and metabolism of arginine, proline, glutamate and asparagine.

We built a network based on the integration of network pharmacology and metabolomics to gain a deeper understanding of the mechanisms of DP in treating DFU. (Figure 10). By importing 114 different metabolites and 108 targets into Cytoscape’s MetScape plug-in, networks of compound-reaction-enzyme-gene were built. Seven critical targets, namely, GSTM1, TYMS, PAH, DDC, DHFR, PGM1 and ABAT, were found by matching the genes in MetScape analysis with the potential targets found in the network pharmacology (Table 2). The key metabolites were Glycine; Thymine; Thymidine; Cytidine 5′-monophosphate (CMP); Uridine; 4-(2-Aminoethyl),2-benzenediol; D-Glucosamine 6-phosphate; N-Acetyineuraminate; and Imidazole-4-acetate. These were nine significant metabolites. The affected pathways were as follows: Leukotriene and Pyrimidine metabolism; Vitamin B9 (folate) metabolism; Urea cycle and metabolism of arginine, proline, glutamate and asparagine; Tyrosine metabolism, Amino sugar metabolism; and Histidine metabolism. They might be key players in how DP treated DFU therapeutically. Combined with network pharmacology and metabonomics, PAH, DHFR, GSTM1 and CAT were found to be the key genes.

**TABLE 2:** The information of key targets, metabolites and pathways.

Related pathway	Key target	Key metabolite
Leukotriene metabolism	GSTM1	Glycine
Pyrimidine metabolism	TYMS	Thymine, Thymidine, CMP, Uridine
Tyrosine metabolism	GSTM1, PAH, DDC	4-(2-Aminoethyl)-1,2-benzenediol
Urea cycle and metabolism of arginine, proline, glutamate and asparagine	GSTM1	Glycine
VitaminB9 (folate)metabolism	DHFR, TYMS	Glycine; 5,10-Methenyltetrahydrofolate
Amino sugars metabolism	PGM1	D-Glucosamine 6-phosphate; N-Acetyineuraminate
Histidine metabolism	ABAT; DDC	Imidazole-4-acetate

### 3.7 Molecular docking analysis

**FIGURE 11:**
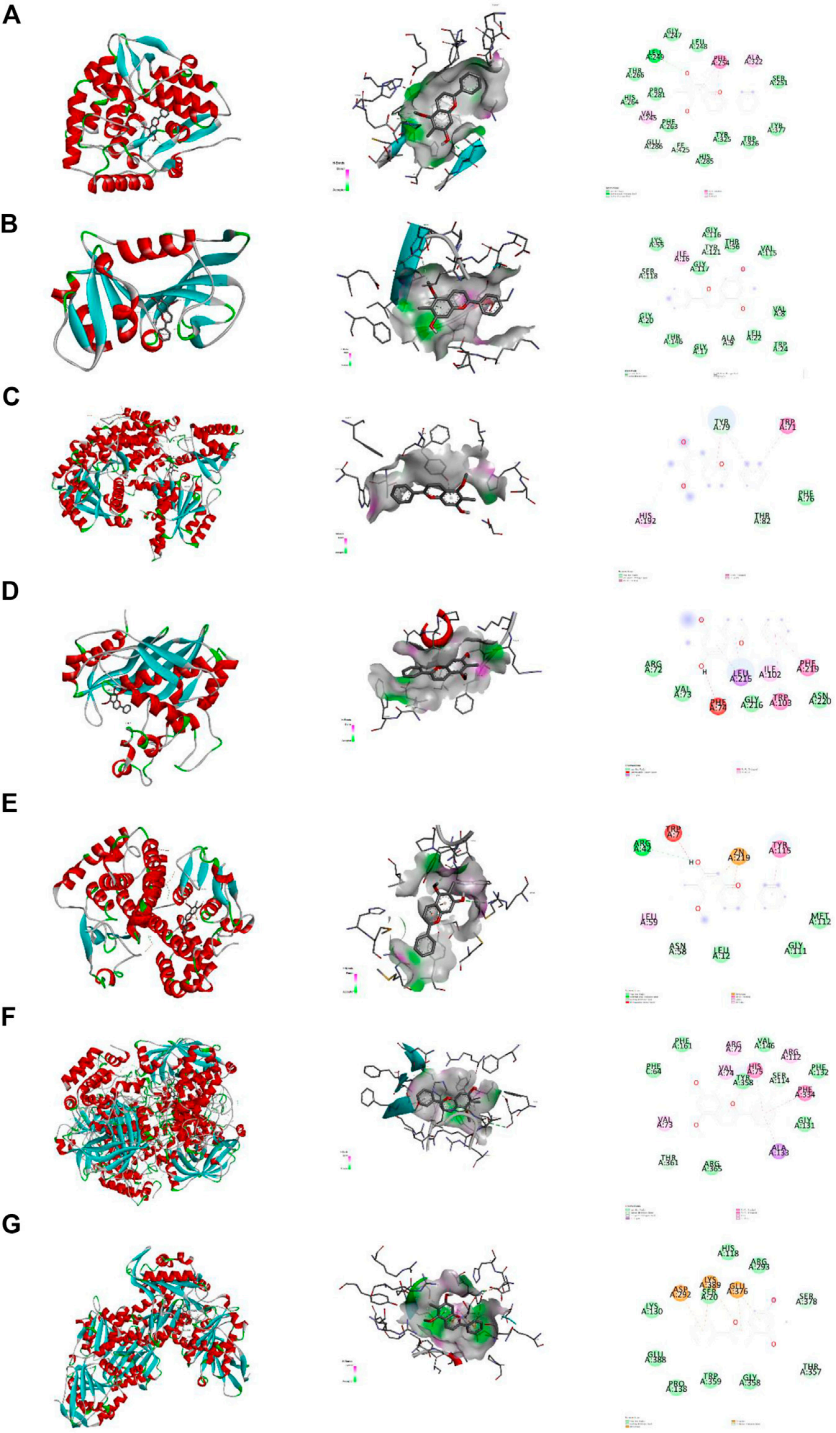
Molecular docking. Note. Binding mode of proteins and ligands. **(A)** Binding mode of GSTM1 with DP. **(B)** Binding mode of TYMS with DP. **(C)** Binding mode of PAH with DP. **(D)** Binding mode of DDC with DP. **(E)** Binding mode of DHFR with DP. **(F)** Binding mode of CAT with DP. **(G)** Binding mode of PGM1 with DP.

Molecular docking was used explore interaction between key targets and DP. With molecular docking, the binding energies of DP with PAH, DHFR, TYMS, DDC, GSTM1, CAT and PGM1 were −8.7 (RMSD:1.105), −7.9 (RMSD: 1.952), −7.3 (RMSD: 1.345), −5.3 (RMSD: 0.886), −5.2 (RMSD: 1.753), −9.6 (RMSD:1.710) and −6.5 (RMSD: 1.311) kcal/mol, respectively. The docking results revealed that DP had a high affinity for the key targets, particularly PAH, DHFR GSTM1 and CAT. Van der Vaals and Pi-Alkyl were the primary forces between the ligand and the receptors (Figure 11).

## 4 Discussion

The wound healing of DFU is challenging. The healing process of a DFU is divided into three main periods, namely, the inflammatory, granulation tissue formation and remodelling stages. Traditional Chinese medicine, Dragon’s blood, can hasten the healing of wounds of DFU rats. It produces anti-inflammatory and antioxidant benefits for the wound (C. Desmarchelier, 1997; Lopes et al., 2004; Gupta et al., 2008). Moreover, it might accelerate epithelial cell differentiation and fibroblast proliferation (A. J. Vaisberg, 1989; Porras-Reyes et al., 1993). In the present research, whether DP, a key component of Dragon’s blood, improves wound healing was explored. Our findings showed that the DP could hasten the wound closure of DFU rats, especially during the inflammatory and granulation tissue formation stages. It inhibits the inflammatory response in the DFU during the inflammatory stage and stimulates collagen deposition to promote wound healing in the granulation tissue formation stage. Furthermore, this is the first-time study to investigate the mechanisms of DP promoting wound healing in DFU rats through the integration of metabolomics and network pharmacology. DP dressing (multi-layered hydrogel/nanofiber dressing loaded with DP) is a novel dressing for the treatment of chronic skin ulcers. The multilayer grade hydrogel/nanofibre dressing is a cured laminate of electrostatic spinning technology and methacrylate-based gelatin, which has the advantages of high porosity, mutual macropore network connectivity, large specific surface area, strong ability to absorb tissue exudate and suitable biodegradability (Hao et al., 2021). DP dressing has unique advantages in the treatment of DFU, because it eliminates the need for repeated routine dressing changes after application, reduces wound irritation and facilitates wound healing. In addition, gelatine provides the arginine-glycine-aspartate (RGD) peptide sequence; this gives the hydrogel made from this polymer good cell adhesion and higher haemostatic capacity (Hao et al., 2021). These are the innovations of our study. We found that Leukotriene metabolism and Pyrimidine metabolism, Urea cycle and metabolism of arginine and proline were important for DP’s DFU treatment. The PAH, DHFR, GSTM1 and CAT are the key genes of DP for the treatment of DFU.

The inflammatory microenvironment plays an essential role in DFU wound repair. In chronic wounds, the inflammation phase is significantly prolonged or even prevented from transitioning into the proliferation phase. As a result, macrophages primarily have a pro-inflammatory M1 phenotype, releasing inflammatory cytokines that cause tissue destruction and organ dysfunction. To further evaluate the internal causes of the wound healing process, the contents of TNF-α, IL-1 and hs-CRP were detected in the wound by ELISA assays. Hs-CRP, the acute phase reactant protein, is used to study the association between inflammation and various chronic diseases, such as metabolic and heart diseases (Doumatey et al., 2014). DFU is a chronic wound, and the sustained high level of hs-CRP is among the factors of wound ischemia; this is not conducive to wound healing. As a well-known pro-inflammatory cytokine, TNF-α is produced by macrophages and monocytes, which are involved in the inflammatory response and immune response. It is a type of polypeptide cytokine secreted by monocyte-macrophages with a variety of biological activities related to the acute phase response, collective immune response and inflammation (Vandekerckhove, 1991).

IL-1 is involved in various inflammatory phenomena, metabolism and cell repair. In addition, as an internal release hormone, it can stimulate the systemic inflammatory response, make the body show a stress state, regulate the cells in the immune system and is closely related to wound repair (Wang, 1997). Several studies showed the proliferation and differentiation of fibroblasts could be influenced by the discharge and secretion of inflammatory cytokines, including IL-1 and TNF-α (P. Qian, 2003). According to Figures 4G–I, the levels of TNF-α, IL-1 and hs-CRP decreased in the Adm group compared with the Wmg and Mod groups. This research demonstrates that DP could enhance wound closer by suppressing inflammation. Topical administration of DP treatment to DFU wounds sustained low inflammation levels, which could effectively stop the inflammatory stage in hyperglycaemic wound tissue from being prolonged. This is consistent with the study of Jiang et al. (2017). Some researchers discovered that Dragon’s blood, as a traditional Chinese medicine, has a noticeable effect on wounds, ulcers, inflammation, diarrhoea and cancer (Z.-P. Chen, 1994; L. Pieters and Berghe, 1995). However, the effect of DP on wound closure has received little attention.

The field of metabolomics is becoming increasingly popular among researchers as a tool for investigating illness processes and potential treatment options. We discovered nine important metabolites and associated pathways of DP in the treatment of DFU. Nevertheless, teamwork is necessary for data analysis and interpretation in the field of metabolomics because of its complexity and heterogeneity (Eicher et al., 2020). Network pharmacology optimised the screening of metabolites of DP in treating DFU and revealed its mechanisms of action. Using a methodology that considers both metabolomics and network pharmacology, we found eight key targets (GSTM1, TYMS, PAH, DDC, DHFR, PGM1, ABAT and CAT), nine key metabolites (Glycine; Thymine; Thymidine; CMP; Uridine; 4-(2-Aminoethyl),2-benzenediol; D-Glucosamine 6-phosphate; N-Acetyineuraminate; and Imidazole-4-acetate) and seven related pathways (Leukotriene metabolism; Vitamin B9 (folate) metabolism; Pyrimidine metabolism; Amino sugars metabolism; Tyrosine metabolism; Urea cycle and metabolism of arginine, proline, glutamate and asparagine; and Histidine metabolism). This strategy provides an appropriate method for validating the results of the two approaches. Screening for metabolites and targets in other natural compounds is also feasible.

DP is a man-made synthetic analogue of dracorhodin. It is a popular choice for use as a high-performance liquid chromatography (HPLC) standard and for researching the biological functions of dracorhodin. It possesses wound healing action on rats, *in vitro* angiogenic activity on HUVEC cells and *in vivo* angiogenic activity on zebrafish embryos (Jiang et al., 2017; Krishnaraj et al., 2019). Previous studies have investigated the possible pathways that could be involved when using DP to treat DFU. According to the findings of some research, DP sped up the process of skin wound healing in DFU rats by regulating the expression of pro-inflammatory cytokines that were triggered by the TLR4 pathway (Li et al., 2016). Additionally, experts elaborated that DP accelerates wound healing by increasing late wound NO levels and eNOS protein expression. Furthermore, according to Jiang X report, DP regulates fibroblast proliferation to expedite rat wound healing (Jiang et al., 2018), which is consistent with this research. Zhang et al. (2014). Found that DP significantly inhibited cell proliferation. It also caused cell cycle arrest and apoptosis in fibroblasts, at least in part by modulating caspase-3 expression and activity, indicating that DP is a feasible choice for hypertrophic scar treatment. DP has been shown by Jiang et al. (2017) to inhibit TNF-α and IL-1β secretions, thereby reducing inflammation. It stimulates TGF and VEGF protein expression, collagen deposition and microvessel formation and then promotes wound closure. He (2011) reported that the therapeutic effect of dragon’s blood on diabetes ulcers was better than that of the insulin in the insulin control group, and the down-regulation of Smad3 and Smad4 expression was the mechanism involved in the promotion of wound healing. In this study, we researched the metabolic changes in the skin tissue of DFU rats. The higher significant number of metabolites and pathways affected by DFU in the skin tissue and the more complex network of interactions are evidence of a more severe metabolic disturbance in DFU rats.

Metabolomics research is limited to metabolites and pathways; no deeper investigation in their interactions has been conducted. Network pharmacology is a method based on bioinformatics and systems pharmacology (Zhong et al., 2018), which assess drug polypharmacological effects at the molecular level to investigate the interaction between natural products and targets and confirm major mechanisms (Sheng et al., 2014). Network pharmacology can help investigate reaction networks and key targets and metabolites (Yu et al., 2012). This integrated strategy discovers the crucial targets and important mechanisms of DP treating DFU rat by integrating network pharmacology and metabolomics.

Arginine is important in cellular physiology. Like other amino acids, it plays a role in the production of proteins. The conversion of arginine to nitric oxide and other polyamines has a role in cell signalling and cell proliferation. Arginine is an essential substrate for wound healing processes due to its multiple functions. In numerous studies, supplying arginine alleviates or improves healing (Witte, 2003). A previous study suggested that arginine metabolite-nitric oxide played a key role in wound healing. As a semi-essential amino acid, arginine is metabolised by arginase and nitric oxide synthase. Wound-healing emphasises the important role of strict reciprocal control among these enzymes (Stechmiller, 2008).

Arginase signalling plays a vital role in chronic wound pathophysiology and healing. As an evolutionarily conserved enzyme, arginase (ARG) can be expressed in a variety of cells. In the last stage of the urea cycle, arginine protects excess ammonia under homeostatic conditions by producing L-ornithine and urea. L-ornithine is located at the intersection of Arg dependent pathway and urea cycle, contributing to collagen production, cell proliferation and detoxification (Szondi et al., 2021). Collagen is an important component of connective tissue; thus, healing requires collagen formation and deposition (B. Behm, 2012). L-proline is an important collagen building block (Shih et al., 2010). Raised synthesis of proline, a component of collagen, leads to post-traumatic increases in wound tensile strength, and wound hydroxyproline levels are increased by parenteral L-arginine injection in both normal and diabetic Lewis rats (Witte, 2003; Caldwell et al., 2018). According to this study, the density of collagen content in Adm group was higher than of Wmg and Mod groups, indicating that DP promoted proline secretion during wound healing. We speculate that DP promotes the production of more arginine and proline in skin tissue to improve the wound and then promotes collagen synthesis and deposition.

Oxidative stress is a major resource of the inflammatory response, and the markers of oxidative stress, such as catalase (CAT) and plasma total antioxidants, play a significant role in non-healing of DFUs. Vairamon et al. discovered factors that influence foot ulcer healing in type 2 diabetics. This research focused on oxidative stress, which is the cause of inflammation. Researchers examined oxidative stress markers in blood samples, such as lipid peroxidation, CAT and others. Catalase levels were higher in subjects with neuroischemic noninfectious ulcers than those with neuroischemic infectious ulcers (*p* < 0.001). The total antioxidant status in plasma gradually decreased in subjects from uninfected ulcer to ischemic infection (*p* < 0.0001). We concluded that level of lipid peroxidation in plasma was high. The decrease of total antioxidant status and the deficiency of antioxidant enzymes are the main reasons for the prolonged inflammatory reaction and chronic ulcer. In type 2 diabetes patients, oxidative stress may be one of the factors leading to the nonhealing of diabetes foot ulcers (Viswanathan, 2010). Based on our study, DP treatment reduced the inflammatory response compared with the control group. This result implied that DP shields organisms from oxidative stress. According to the network pharmacology analysis, the mechanism may involve CAT, which activates metabolic pathways. The topical application of DP increased the activity of antioxidant enzymes such as CAT, which promotes wound healing. Molecular docking also showed that DP and CAT are well combined. In DFU, where tissue inflammation and oxidative stress were elevated primarily due to an increase in reactive oxygen species (ROS) (Busik et al., 2008), the topical administration of DP alleviates the ROS insults, thus protecting the wound skin tissue from oxidative injuries.

The enzyme dihydrofolate reductase (DHFR) is a validated target for several antimicrobials. Meanwhile, it is necessary for the *de novo* biosynthesis of folate species. Gustavo P. ribodi et al. investigated the structural and biochemical properties of *Mycoplasma ulcerum* DHFR, and explored its interaction with P218. This research revealed the P218 could be further developed as a therapeutic strategy for Buruli ulcer (Riboldi et al., 2021). Shailesh K et al. discovered *Klebsiella pneumoniae* containing DHFR in eight out of (18.18%) of 44 different DFU patients. Based on case analysis, they proposed a great need to develop a treatment that inhibits the growth of multidrug-resistant *K. pneumoniae* in DFU patients. Simultaneously, it can reduce or even eliminate the risk of amputation (Shahi et al., 2013). GutGards has activity of anti-*Helicobacter pylori* through inhibiting DNA gyrase, protein synthesis and DHFR, according to Mannanthendil Kumaran Asha et al., to treat peptic ulcer disease (Asha et al., 2013). In this study, the molecular docking binding energy of DP and DHFR was −7.9 kcal/mol. We hypothesised that DP might exert anti-inflammatory activity by inhibiting DHFR in the treatment of wound skin ulcers.

The nucleophilic assault of reduced glutathione (GSH) on electrophiles or other compounds that are attracted to electrons is facilitated by glutathione s-transferases (GSTs). GSTs occupy important positions in two ways, as follows: the binding of glutathione and endogenous products of lipid peroxidation; and protecting cells from harmful oxidative stress. GSTM1 and GSTT1, two isoforms of GST with functional polymorphisms, have been investigated in many chronic diseases. Romério Alencar de Oliveira Filho et al. reported that patients with GSTM1 null had 3.9 times higher risk of stroke, and a high risk of acute chest syndrome or malleolar ulcers (OR = 4.2 and 6.9, respectively). GSTM1 null genotypes had a high risk of developing malleolar ulcer, acute chest syndrome and femoral head aseptic necrosis (de Oliveira Filho et al., 2013). By combining metabolomics and network pharmacology, we created a compound-enzyme-reaction-gene network and detected that the hub target, GSTM1, was involved in the metabolic pathway response of DFU rats treated with DP. Molecular docking showed a better binding ability of DP and GSTM1.

The liver produces phenylalanine hydroxylase (PAH), an amino acid metabolic enzyme that catalyses the reaction of phenylalanine to tyrosine, thus participating in the gluconeogenesis process. In diabetes, the activity of this enzyme is obviously increased, gluconeogenesis is accelerated, and blood sugar is increased (Liang., 1996). In this study, we discovered that compared with the Mod group, blood glucose of Adm group decreased significantly after DP treatment. Further molecular docking analysis (the binding energy of DP and PAH is −8.7 kcal/mol) indicated that the mechanisms of DP’s promotion of wound closure in DFU rats may be through the inhibition of the activity of PAH, thereby exerting a hypoglycaemic effect.

Tyrosine metabolism is also implicated in DFU. The concentration of L-tyrosine is significantly elevated in DFU patients. This is fatal in the chronic state and can lead to amputation in severe cases (Roy, 2020). The Mod group had more L-tyrosine than that of the Con group, which may have a negative impact on wound healing. Bonner-Weir S, 1983, Liang, 1996, Lin, 2018.

**FIGURE 12:**
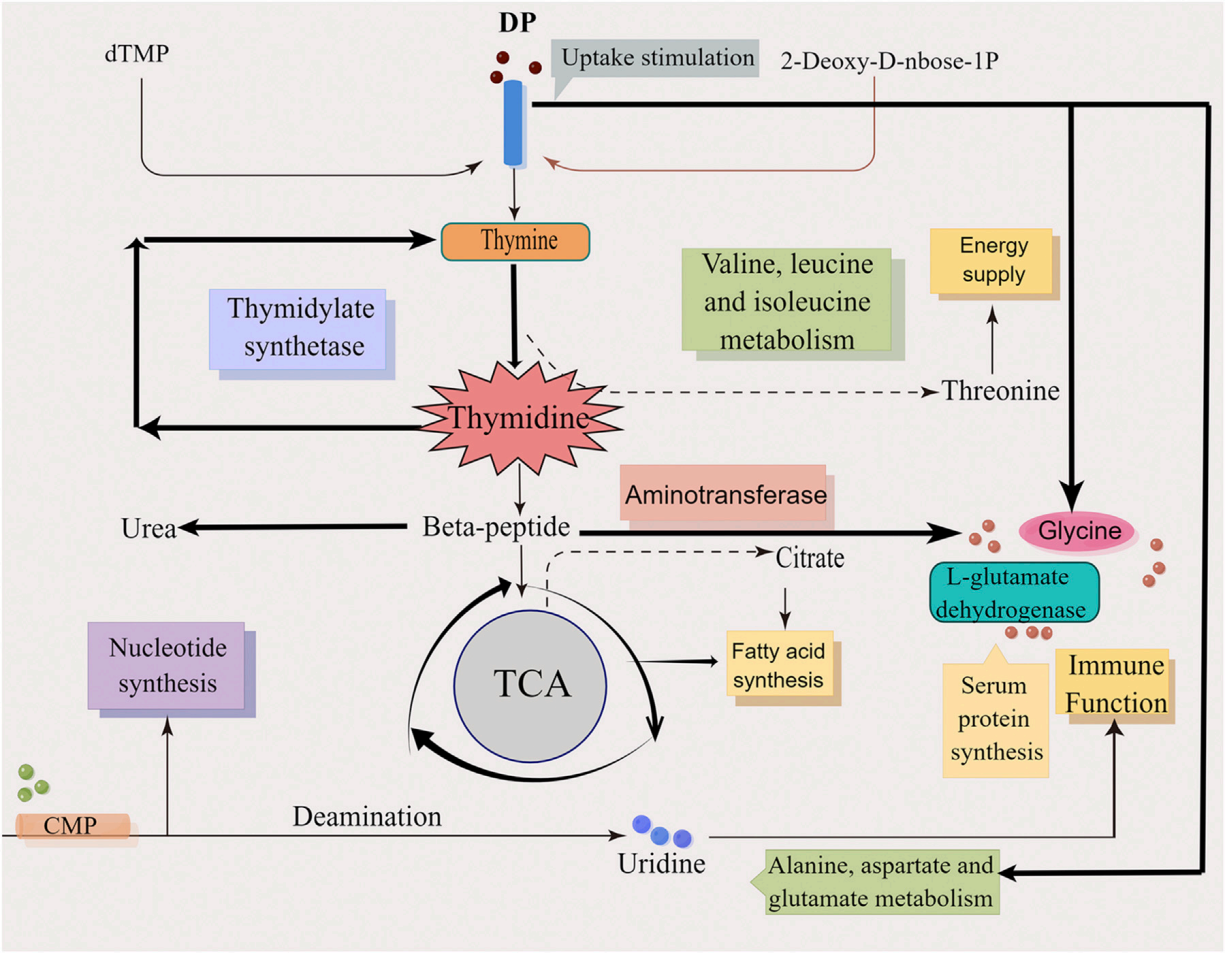
The mechanism diagram of DP in treating DFU rats.

Collectively, the main action mechanisms for treating DFU by DP was attributed to the regulation of pyrimidine metabolism and alanine, aspartate and glutamate metabolism and other pathways and metabolites, as well as the regulation of protein synthesis, energy supply, immune function and other biological processes that promote wound healing (Figure 12).

However, this study has some limitations. We still need to verify the precise mechanisms *via* systematic molecular biology experiments. For example, this study will be more perfect if WB and Q-PCR were added to detect the expression of important targets in different groups and proteomics and transcriptomics are used to further explore the mechanism of DP treatment of DFU at different levels.

## 5 Conclusion

In this study, we used a combined metabolomics and network pharmacology approach for the first time to elucidate the mechanisms of DP in treating DFU rats. The topical application of DP promotes wound healing in the DFU rats *via* various mechanisms that result in the maintenance of tissue growth and proliferation at the wound site. By inhibiting inflammation response and oxidative stress, DP may suppress the prolonged inflammatory phase in DFU wounds and stimulate collagen deposition to promote wound healing. The results obtained in this study indicated the core targets and mechanisms of DP in treating DFU rats. In DFU rats, DP could regulate the metabolic response of skin tissue and accelerate wound healing. The integration revealed four core targets with associated metabolites and pathways. Molecular docking was performed to further validate the interaction between targets and DP. Our study provides theoretical and data support for a more in-depth study of the mechanisms of DP in the treatment of DFU and provides an experimental basis for the development of a new type of dressing to promote the wound healing of DFU.

## Data Availability

The datasets presented in this study can be found in online repositories. The names of the repository/repositories and accession number(s) can be found in the article/Supplementary Material.
