# Supplementary material for: Combined metabolomics and network pharmacology to elucidate the mechanisms of Dracorhodin Perchlorate in treating diabetic foot ulcer rats
Source: Front Pharmacol. 2022 Nov 18;13:1038656. doi: 10.3389/fphar.2022.1038656 (PMC9752146; doi:10.3389/fphar.2022.1038656)
Supplement: Supplementary file 4 [file DataSheet1.docx]

**Supplementary Materials**


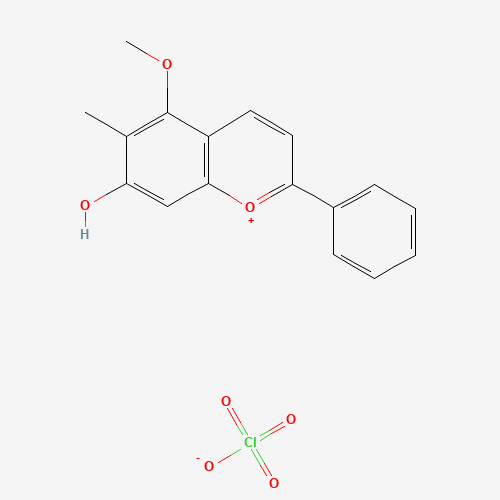


**FIGURE S1│**The chemical structure of Dracorhodin Perchlorate (DP).


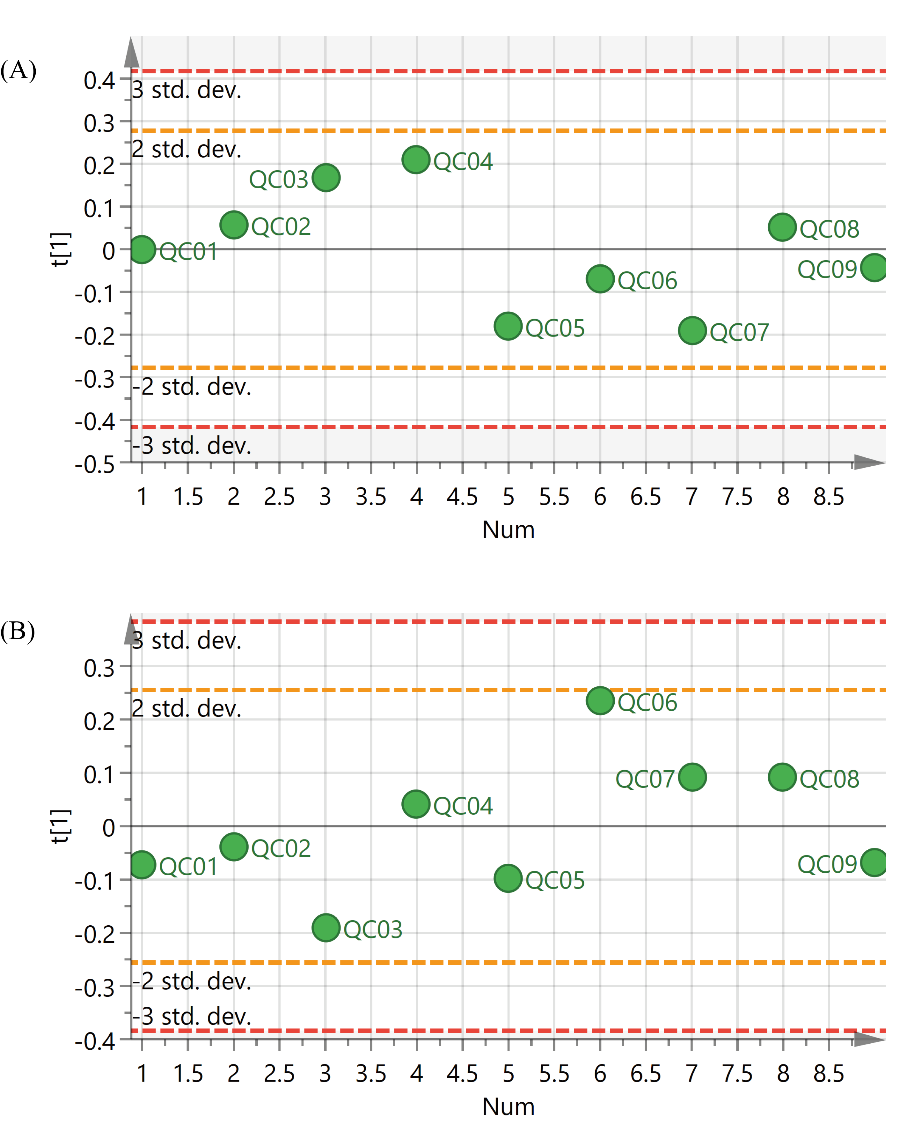


**FIGURE S2│**PCA score plots (A and B) of QC samples for metabolomic validation.


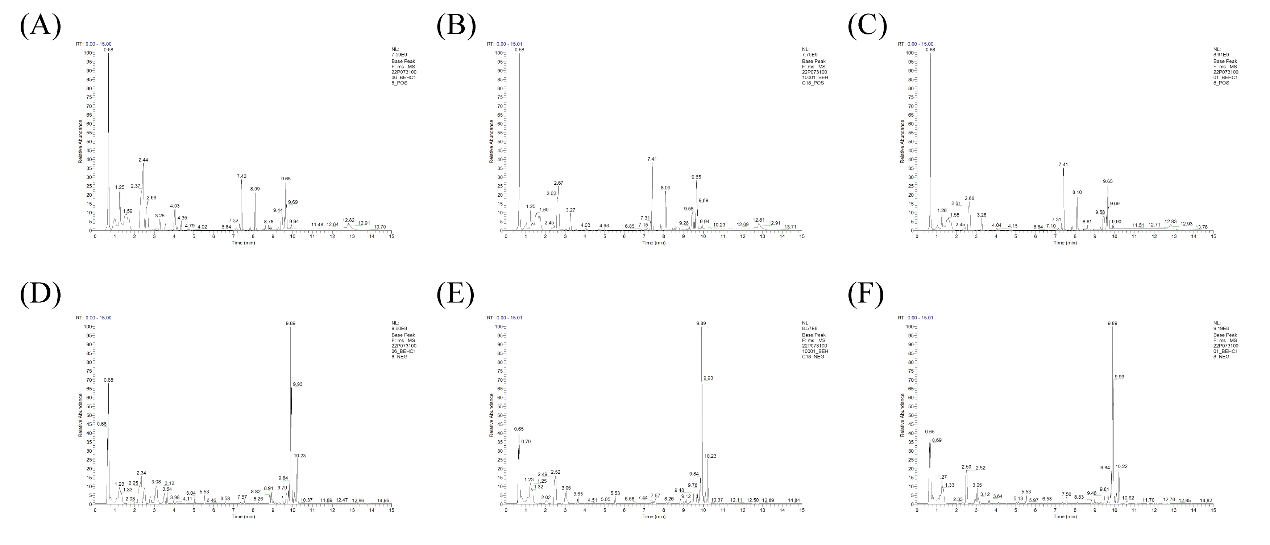


**FIGURE S3│**Typical BPC for each group of samples. (A) (B) (C)is the BPC diagram in pos mode. (A) is Adm group. (B) is Mod group. (C) is Con group. (D) (E) (F) is the BPC diagram in neg mode. (D) is Adm group. (E) is Mod group. (F) is Con group.


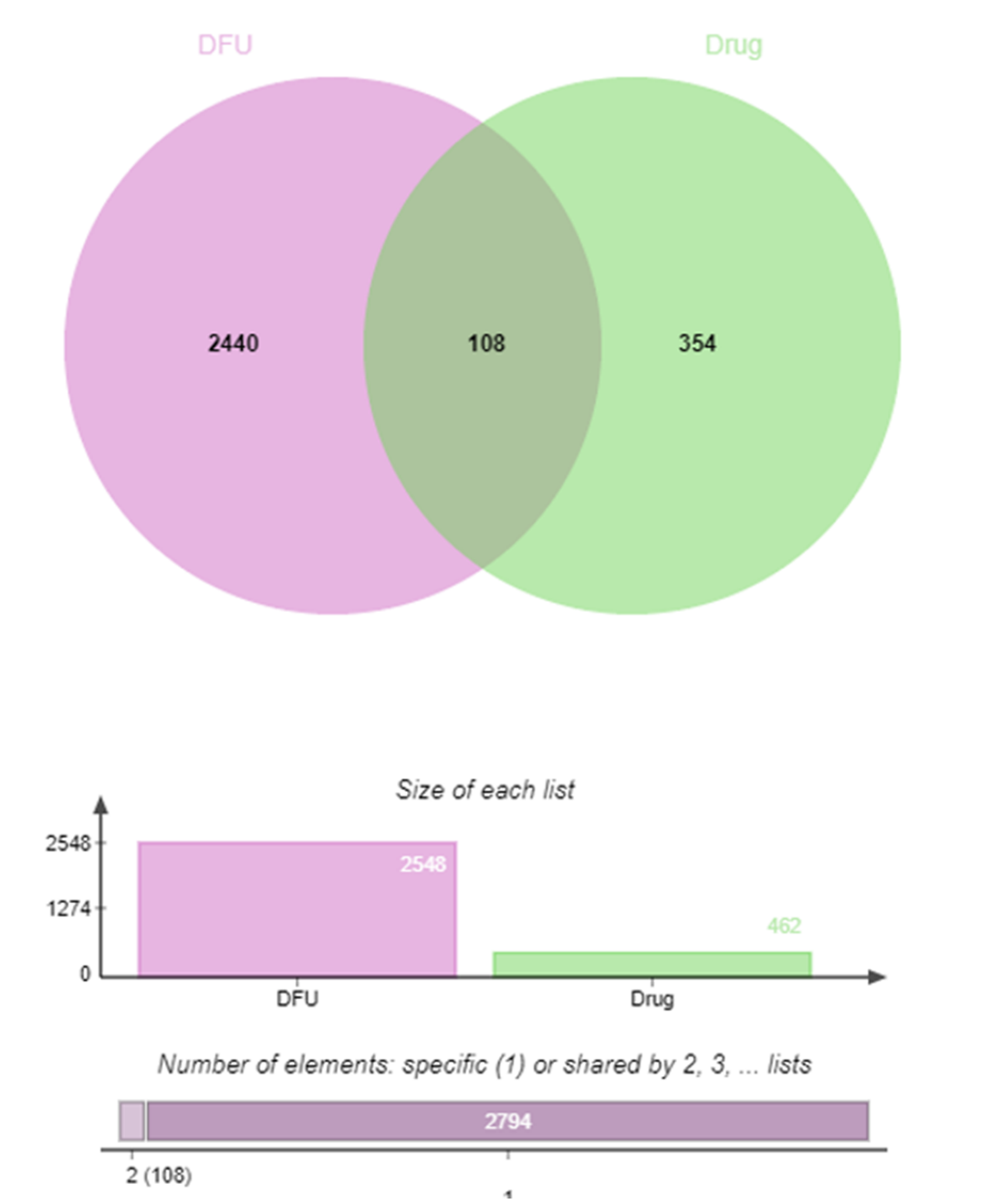


**FIGURE S4│**Venn diagram of 108 common targets related to DP and DFU.

**
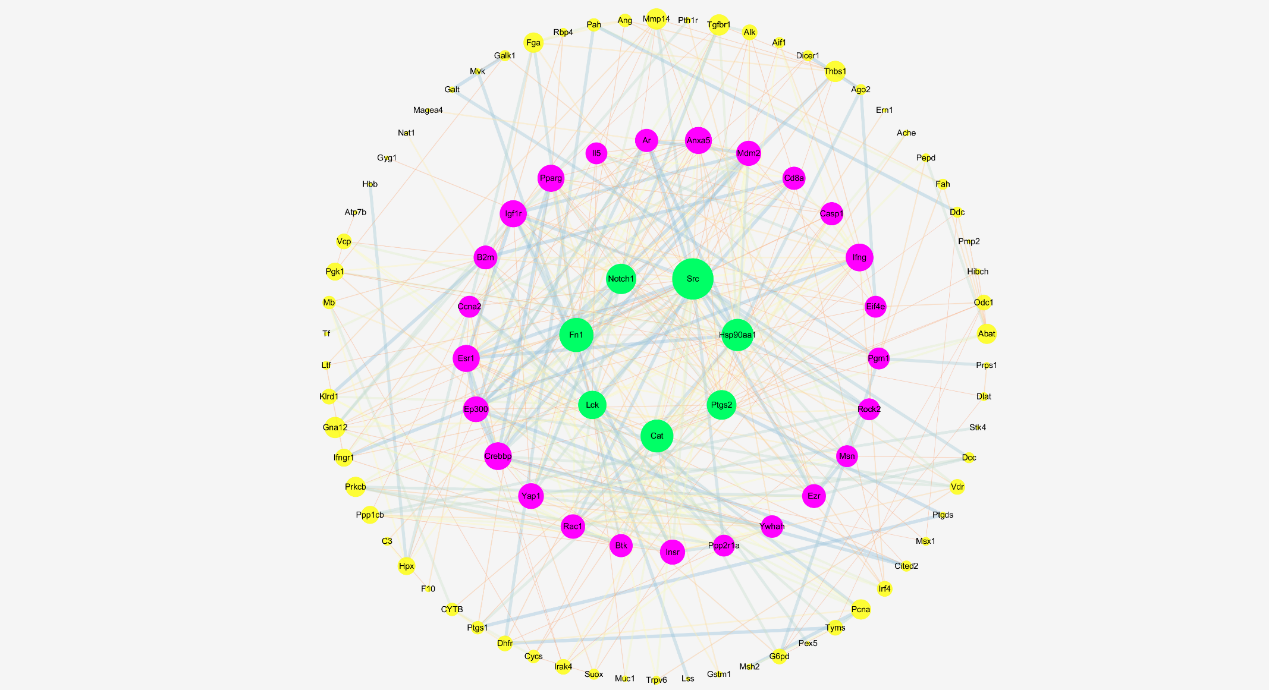
**

**FIGURE S5│**PPI network of DP in treating DFU. The color of a node represents its degree. Hub genes are shown by the nodes with green borders.

**TABLE S1**│The gradient conditions.

| Time | Gradient Condition |
| --- | --- |
| 0-1minute | 98% A; 2% B |
| 1-9 minute | 98%-2% A; 2%-98% B |
| 9-12minute | 2% A; 98% B |
| 12-12.1minute | 2%- 98% A; 98% - 2% B |
| 12.1-15minute | 98% A; 2% B |

| **TABLE S2**│Parameters of molecular docking between DP and targets. | | | | | | |
| --- | --- | --- | --- | --- | --- | --- |
| Targets | center x | center y | center z | size x | size y | size z |
| GSTM1 | 28.2 | 8.8 | 13.4 | 18.3 | 18.4 | 16.6 |
| TYMS | 92.5 | -10.5 | 37.8 | 22.9 | 20 | 27.9 |
| PAH | -4.1 | 25.3 | 4.9 | 18.3 | 11.8 | 14.3 |
| DDC | 56.8 | 27.4 | 16.4 | 22.1 | 14.5 | 18.5 |
| DHFR | 5 | -2.5 | -21.9 | 23.9 | 27.1 | 22 |
| CAT | 25.5 | 44.4 | 69.1 | 24.5 | 31 | 37.9 |
| PGM1 | -17.8 | -46.9 | -25.3 | 14.4 | 12.9 | 18.6 |

| **TABLE S3**│ PLS-DA parameter. | | | | | | |
| --- | --- | --- | --- | --- | --- | --- |
| mode | group | Modelling parameters | | Validation parameters | |  |
|  |  | R2Y(cum) | Q2(cum) | R2 | Q2 |  |
| pos | Mod_Con | 0.99 | 0.85 | (0.0,0.88) | (0.0, -1.18) |  |
| pos | Adm_Mod | 0.99 | 0.6 | (0.0,0.95) | (0.0, -0.89) |  |
| neg | Mod_Con | 1 | 0.92 | (0.0,0.85) | (0.0, -1.25) |  |
| neg | Adm_Mod | 1 | 0.68 | (0.0,0.92) | (0.0, -0.99) |  |
